# Vascular endothelial growth factor as a potential biomarker in systemic sclerosis: a systematic review and meta-analysis

**DOI:** 10.3389/fimmu.2024.1442913

**Published:** 2024-11-28

**Authors:** Angelo Zinellu, Arduino A. Mangoni

**Affiliations:** ^1^ Department of Biomedical Sciences, University of Sassari, Sassari, Italy; ^2^ Discipline of Clinical Pharmacology, College of Medicine and Public Health, Flinders University, Bedford Park, SA, Australia; ^3^ Department of Clinical Pharmacology, Flinders Medical Centre, Southern Adelaide Local Health Network, Bedford Park, SA, Australia

**Keywords:** vascular endothelial growth factor, systemic sclerosis, biomarkers, vascular dysfunction, fibrosis, complications

## Abstract

**Introduction:**

Systemic sclerosis (SSc), a chronic autoimmune condition, is characterized by microvascular dysfunction, ineffective angiogenesis, and fibrosis. The identification of robust biomarkers reflecting these processes may assist in clinical management and lead to the discovery of new therapies. We sought to address this issue by conducting a systematic review and meta-analysis of studies investigating one such biomarker, vascular endothelial growth factor (VEGF), in SSc patients and healthy controls and in SSc patients with localized or diffuse disease, different video capillaroscopy patterns (early, active, or late), and presence or absence of complications.

**Methods:**

We searched PubMed, Scopus, and Web of Science from inception to 15 May 2024. We assessed the risk of bias and the certainty of evidence using the JBI checklist for analytical studies and GRADE, respectively.

**Results:**

In 42 eligible studies, compared to controls, patients with SSc had significantly higher plasma or serum VEGF concentrations (standard mean difference, SMD=0.93, 95% CI 0.71 to 1.15, p<0.001; moderate certainty). In further analyses, VEGF concentrations were significantly higher in SSc patients with diffused disease than those with localized disease (SMD=0.30, 95% CI 0.01 to 0.59, p=0.046; very low certainty), in patients with late vs. active video capillaroscopy pattern (SMD=0.35, 95% CI 0.09 to 0.61, p=0.008; very low certainty), and in patients with pulmonary hypertension than those without (SMD=0.93, 95% CI 0.34 to 1.53, p=0.002; very low certainty). By contrast, no significant differences were observed between SSc patients with and without digital ulcers, interstitial lung disease, and telangiectasias, whereas limited evidence was available for alveolitis. Meta-regression and subgroup analysis of studies investigating VEGF in SSc patients and controls showed no significant associations between the effects size and various patient and study characteristics, including SSc duration and use of corticosteroids, immunosuppressors and vasodilators. By contrast, significant associations were observed with the geographical location where the study was conducted.

**Discussion:**

The results of this systematic review and meta-analysis suggest that VEGF can be useful in the assessment and management of SSc and in the identification of novel therapeutic strategies in this patient group.

**Systematic Review Registration:**

https://www.crd.york.ac.uk/prospero, identifier CRD42024552925.

## Introduction

Systemic sclerosis (SSc) is a chronic and disabling autoimmune condition that is characterized by microvascular dysfunction, ineffective angiogenesis, and localized or diffuse fibrosis (1–4). There is increasing evidence that microvascular damage is a critical pathophysiological step in SSc as it generally occurs before the onset of skin and visceral fibrosis (5, 6). Early clinical manifestations of microvascular damage in SSc primarily involve the Raynaud’s phenomenon, with other manifestations such as telangiectasias, pitting scars, nailfold video capillaroscopy abnormalities, digital ulcers, and pulmonary arterial hypertension occurring during later stages of the disease (7–9). The presence of microvascular offers significant opportunities for the study and the identification of novel SSc biomarkers, an important knowledge gap in this patient population (10, 11). Such biomarkers might facilitate early diagnosis and treatment, critical factors associated with disease progression and clinical outcomes (3, 4, 12). The available evidence suggests that endothelial cell injury secondary to multiple insults, e.g., autoantibodies, viral agents, and excess production of reactive oxygen species, leads to a dysregulation in the production of vasoconstrictive and vasodilating substances, including excess endothelin-1 and reduced nitric oxide (13–17). Alterations in nitric oxide synthesis in SSc patients are also associated with increased concentrations of asymmetric dimethylarginine, an endogenous nitric oxide synthase inhibitor (18, 19). Functional and structural endothelial alterations are also associated with increased expression of cell adhesion molecules and chemokines, which further perpetuates microvascular damage and alterations in vascular tone (20). Overall, these processes lead to a dysregulated increase in pro-angiogenic factors, i.e., vascular endothelial growth factor (VEGF) and endoglin (21, 22), and anti-angiogenic molecules such as pentraxin-3, endostatin, and angiostatin (23, 24). These observations have stimulated a significant body of research to investigate a broad group of potential biomarkers of SSc, including selectins, immunoglobulin-like cell adhesion molecules, VEGF, endoglin, endothelin-1, pentraxin-3, endostatin, angiostatin, angiopoietins, matrix metalloproteinases, neurovascular guidance molecules, sirtuins, cytokines, adipokines, thrombomodulin, soluble CD163, brain natriuretic peptide, von Willebrand factor, and soluble urokinase plasminogen activator receptor (25).

The most studied angiogenic modulator in SSc is VEGF, also known as VEGF-A, the main component of the VEGF family (26). Physiologically, VEGF is a potent pro-angiogenic factor and an essential growth factor for endothelial cells, ensuring the functional and structural integrity of the endothelium and blood vessels through its binding to the target receptors VEGFR-1 and VEFGR-2 as well as non-signaling co-receptors (27). Experimental and clinical studies have reported VEGF activation and increased concentrations in plasma or serum in SSc despite the lack of effective angiogenesis (28–30). Therefore, VEGF activation might further contribute to alterations in blood vessel morphology and tone in SSc (28). This hypothesis is supported by investigations reporting increased VEGF concentrations in SSc patients with systemic fibrosis, specific alterations in nailfold capillary density and patterns (31, 32), and well-established complications, e.g., pulmonary arterial hypertension (31, 33). The significant associations between VEGF elevations, critical pathophysiological processes (microvascular dysfunction, ineffective angiogenesis, and fibrosis) and clinical manifestations suggest that VEGF might represent a useful diagnostic and prognostic biomarker in SSc.

We sought to investigate the potential role of VEGF in SSc by conducting a systematic review and meta-analysis of studies reporting VEGF concentrations in SSc patients and healthy controls and in SSc patients with specific disease types (localized or diffuse), nailfold video capillaroscopy patterns (early, active, or late) (34), and complications. We also investigated associations between the effect size of the differences in VEGF concentrations and specific study and patient characteristics.

## Materials and methods

### Literature search and study selection

We conducted a systematic search in electronic databases (PubMed, Web of Science, and Scopus) from inception to 15 May 2024, using the following terms: “systemic sclerosis” OR “scleroderma” AND “VEGF” OR “vascular endothelial growth factor”. Two investigators independently screened each abstract and, if relevant, the full text articles. Inclusion criteria were: (i) the investigation of VEGF concentrations in patients with SSc diagnosed according accepted guidelines and healthy controls in a case-control study, (ii) evaluation of VEGF concentrations in relation to disease type (localized or diffuse) and/or video capillaroscopy pattern (early, active, or late), (iii) assessment of VEGF concentrations in SSc patients with or without specific complications, (iv) inclusion of adult participants, and (v) availability of the full text of the article in English language. Exclusion criteria were: (i) investigation of VEGF concentrations in immunological conditions other than SSc, (ii) inclusion of participants under 18 years, and (iii) study design other than case-control.

The investigators independently hand-searched the references of the retrieved articles to identify additional studies, and extracted the following variables from each article: year of publication, first author, country and continent where the study was conducted, number of participants, age, male-to-female ratio, mean disease duration, VEGF concentrations, biological matrix assessed (serum or plasma), use of glucocorticoids, immunosuppressors, and vasodilators, fraction of patients affected by diffuse or localized form, early, active, or late video capillaroscopy patterns, digital ulcers, pulmonary hypertension, interstitial lung disease, telangiectasias, and alveolitis.

We assessed the risk of bias using the Joanna Briggs Institute (JBI) Critical Appraisal Checklist for analytical studies (35), and the certainty of evidence using the Grades of Recommendation, Assessment, Development, and Evaluation (GRADE) Working Group system (36). We fully adhered to the PRISMA 2020 statement (**Supplementary Table 1**) (37), and registered the study protocol in an international repository (PROSPERO registration number: CRD42024552925).

### Statistical analysis

We calculated the standardized mean differences (SMDs) and 95% confidence intervals (CIs) for each study to generate forest plots to investigate differences in VEGF concentrations between SSc patients and healthy controls and between SSc with different disease type, video capillaroscopy pattern, and with or without complications. A p-value <0.05 was considered statistically significant. We extracted data from graphs using the Graph Data Extractor software (San Diego, CA, USA) and extrapolated means and standard deviations from medians and interquartile or full ranges as previously reported (38). SMD heterogeneity was assessed using the Q statistic (significance level at p<0.10) and ranked as low (I^2^ ≤25%), moderate (25%< I^2^ <75%), or high (I^2^ ≥75%). We used a random-effects model based on the inverse-variance method in presence of high heterogeneity (39, 40).

We conducted sensitivity analyses to confirm the stability of the results (41), and assessed the presence of publication bias using standard methods (42–44). We also conducted univariate meta-regression and subgroup analyses to investigate possible associations between the effect size and the following parameters: year of publication, study country and continent, number of participants, age, male-to-female ratio, mean disease duration, sample matrix (serum or plasma), disease type, video capillaroscopy pattern, complications, and use of glucocorticoids, immunosuppressors, or vasodilators (45, 46). Statistical analyses were performed using Stata 14 (Stata Corp., College Station, TX, USA).

## Results

### Systematic search and study selection

**Figure 1:**
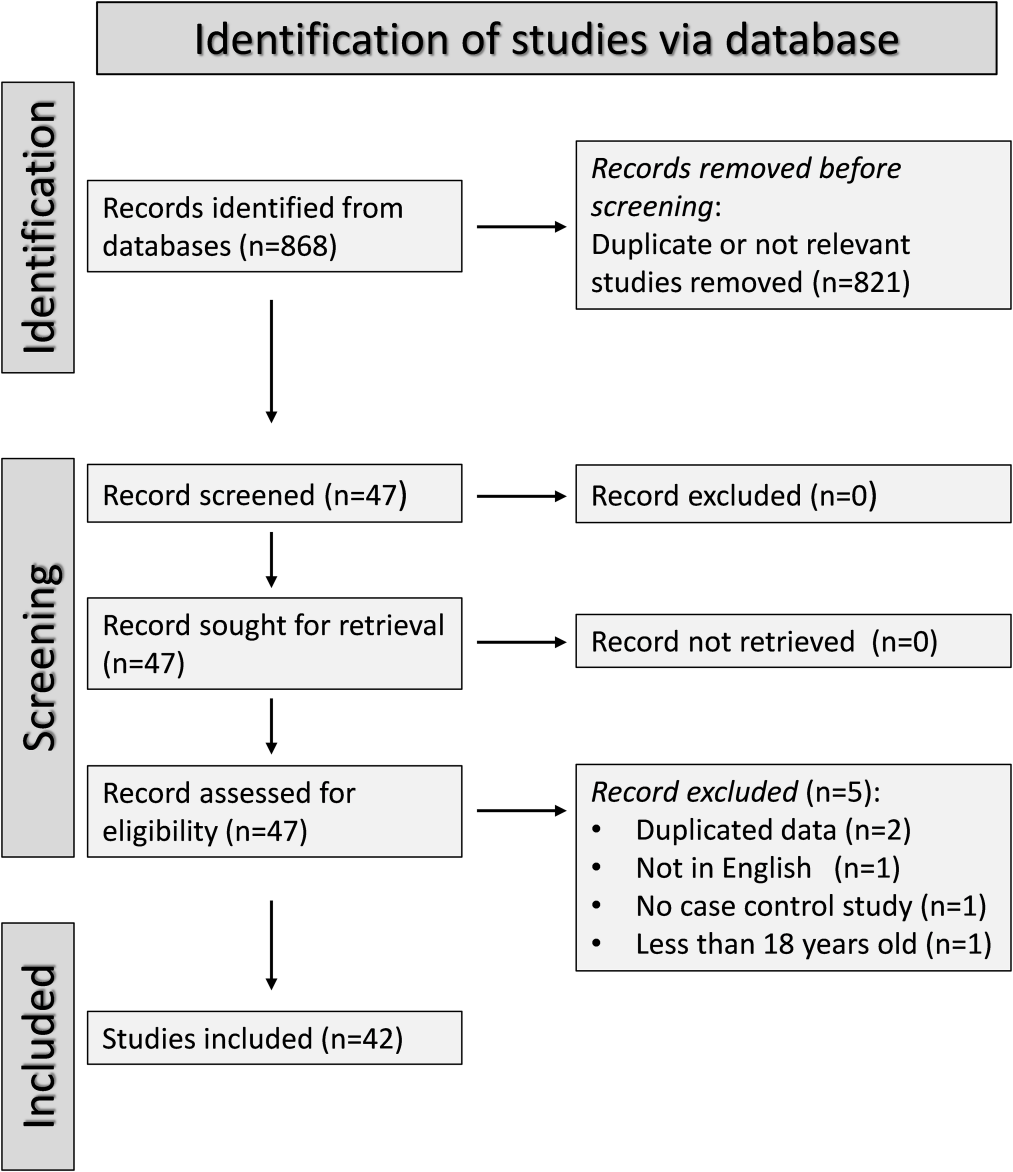
PRISMA 2020 flow diagram of study selection.

The flow chart of the screening process is illustrated in **Figure 1**. After initially identifying 568 articles, 521 were excluded because they were either irrelevant (i.e., different biological matrices analysed such as urine or tissues, cellular or molecular studies, animal studies, pharmacological trials outside the scope of our systematic review, longitudinal studies without control groups, and studies without a case-control or cohort design), or presented duplicate data. Full-text review of the remaining 47 articles led to the further exclusion of two studies because they presented duplicate data, one study because it was not case-control, one study written in a non-English language, and one study including participants under 18 years. Therefore, 42 studies were included in the final analysis (22, 28, 31–33, 43, 47–82). The risk of bias was low in 29 studies (28, 32, 47–51, 53–56, 58, 59, 62–65, 68–72, 74–79, 81) and moderate in the remaining 13 (22, 31, 33, 43, 52, 57, 60, 61, 66, 67, 73, 80, 82) (**Supplementary Table 2**). The initial level of certainty was adjudicated as low (level 2) given the case-control design of the selected studies.

### Presence of SSc

Thirty-eight studies including 39 group comparators assessed VEGF concentrations in 2,181 SSc patients (mean age 52 years, 87% females) and 1,065 healthy controls (mean age 48 years, 82% females) (22, 28, 31–33, 43, 47–52, 54–58, 60–63, 66–82) (**Table 1**). Twenty studies were conducted in Europe (22, 28, 32, 33, 43, 47, 49, 51, 52, 54, 55, 58, 68, 71, 72, 74, 75, 79–81), 14 in Asia (31, 48, 50, 60, 61, 66, 67, 69, 70, 73, 76–78, 82), three in Africa (56, 57, 63), and one in America (62). VEGF was measured using an enzyme-linked immunosorbent assay in 32 studies (22, 28, 31–33, 47–51, 54–58, 60–63, 66, 67, 69, 71–74, 76–81) and a platform for multi-analyte profiling in the remaining six (43, 52, 68, 70, 75, 82). Thirty-two studies measured VEGF in serum (22, 28, 31–33, 43, 47, 49–52, 54–56, 58, 60, 61, 63, 66–68, 71, 72, 74, 76–79, 81, 82) and eight in plasma (48, 57, 62, 69, 70, 73, 75, 80). Disease duration was reported in 24 studies and ranged between 1.7 and 17.25 years (22, 31–33, 47, 48, 50–52, 55, 56, 62, 63, 67–71, 73–78).

**Table 1:** Main characteristics and results of the studies included in the meta-analysis.

		Healthy controls				Patients with systemic sclerosis				
Study	Samplematrix	n	Age (Years)	M/F	VEGF (Mean ± SD)	n	Age (Years)	M/F	VEGF (Mean ± SD)	MDD (Years)
Harada M et al., 1998, Japan (60)	S	10	40	6/4	74 ± 32	13	53	8/5	104 ± 87	NR
Kikuchi K et al., 1998, Japan (66)	S	20	50	4/16	184 ± 62	40	53	3/37	271 ± 195	NR
Sato S et al., 2001, Japan (77)	S	20	matched	matched	182.5 ± 282.1	32	47	3/29	268.9 ± 256.8	5.8
Distler O et al., 2002, Italy (28)	S	21	58.8	5/16	145 ± 100	43	56.3	8/35	517 ± 241	NR
Choi JJ et al., 2003, Korea (31)	S	30	38	0/30	91 ± 64	48	40.6	3/45	264 ± 333	6.9
Hashimoto N et al., 2003, Japan (61)	S	11	NR	NR	61 ± 33	32	52	3/29	356 ± 243	NR
Allanore Y et al., 2004, France (47)	S	20	51	3/17	240 ± 128	40	57	7/33	772 ± 438	6
Kuwana M et al., 2004, Japan (69)	P	11	52.7	0/11	12 ± 5.2	11	57.7	0/11	32.9 ± 35.7	9.33
Kuryliszyn-Moskal A et al., 2005, Poland (33)	S	30	matched	matched	172.4 ± 78.2	31	55.2	0/31	273.2 ± 145.8	7.8
Dziankowska-Bartkowiak B et al., 2006, Poland (55)	S	20	45.3	5/15	326 ± 183	28	45.8	6/22	286 ± 207	6.25
Wipff J et al., 2008, France (22)	S	48	59.4	8/40	261.2 ± 108.6	187	55.9	30/157	445.5 ± 295.5	8.1
Hummers LK et al., 2009, USA (62)	P	27	57.5	10/17	26.1 ± 22.4	113	53	13/100	163.5 ± 176.4	9.6
Papaioannou AI et al., 2009, Greece (72)	S	13	55.3	3/10	196 ± 49	40	56.75	7/33	294 ± 122	NR
Solanilla A et al., 2009, France (80)	P	20	matched	matched	44 ± 31	40	matched	matched	293 ± 126	NR
Distler JHW et al., 2011, Switzerland (54)	S	66	42.7	22/44	152 ± 153	40	47	4/36	376 ± 501	NR
Riccieri V et al., 2011, Italy (75)	P	16	matched	matched	206 ± 145	65	52.7	2/63	383 ± 213	9.63
Avouac J et al., 2013, France (32)	S	20	matched	matched	377 ± 155	60	54	14/46	706 ± 304	17.25
Aydoğdu E et al., 2013, Turkey (48)	P	20	49.3	1/19	595.17 ± 389.4	40	48.35	2/38	619.04 ± 419.8	10.9
Farouk HM et al., 2013, Egypt (57)	P	20	38.9	3/17	38.6 ± 14.57	25	40.3	4/21	106.48 ± 50.2	NR
Koca SS et al., 2014, Turkey (67)	S	28	42.5	6/22	330.9 ± 195.6	37	45.7	5/32	337.4 ± 242.2	4.2
Reiseter S et al., 2015, Norway (74)	S	100	NR	NR	150.4 ± 107	298	56	55/243	209 ± 150	4
Silva I et al., 2015, Portugal (79)	S	34	matched	matched	167 ± 93	77	52.9	5/72	383 ± 297	NR
Cossu M et al. (a) 2016, Italy (52)	S	43	NR	NR	59.22 ± 32.99	95	57.4	NR	74.99 ± 39.55	4.4
Cossu M et al. (b) 2016, Italy (52)	S	43	NR	NR	59.22 ± 32.99	86	59	NR	81.34 ± 49.21	13
Park JK et al., 2016, Korea (73)	P	14	NR	NR	54.2 ± 24.6	26	53.6	1/25	115 ± 53.7	11.6
Yalçınkaya Y et al., 2016, Turkey (82)	S	20	matched	matched	704 ± 363	72	44.9	6/66	776 ± 591	NR
Benyamine A et al., 2017, France (49)	S	41	56.1	3/38	48.9 ± 40.5	45	61.5	1/44	71.3 ± 60.5	NR
Shenavandeh S et al., 2017, Iran (78)	S	44	39.4	3/41	93.9 ± 25.2	44	40.7	4/40	363.4 ± 133.9	4.68
Ibrahim SE et al., 2018, Egypt (63)	S	35	29.8	NR	83.17 ± 3.88	35	30.4	2/33	118.8 ± 28.84	1.7
Saranya C et al., 2018, India (76)	S	30	38	0/30	184 ± 47	55	39	0/55	663 ± 400	2
Michalska-Jakubus M et al., 2019, Poland (71)	S	27	52.4	0/27	233.9 ± 138.3	47	56.4	0/47	329.44 ± 245.16	9.99
Gigante A et al., 2020, Italy (58)	S	10	51.9	2/8	139 ± 87.5	55	53.2	9/46	240.3 ± 149.5	NR
LV T et al., 2020, China (70)	P	15	matched	matched	88 ± 29	30	44	12/18	105 ± 15	4.5
Waszczykowska A et al., 2020, Poland (81)	S	25	59.4	5/20	197.74 ± 155.04	25	57.1	4/21	346.27 ± 399.88	NR
El Gharbawy NH et al., 2021, Egypt (56)	S	20	48.9	NR	1530 ± 437	30	49.3	NR	3445.9 ± 1183.5	9.9
Stern EP et al., 2021, UK (43)	S	12	34	NR	13 ± 19	40	57	NR	20 ± 18	NR
Bhattacharjee D et al., 2023, India (50)	S	20	35.3	4/16	3460 ± 3970	56	35.4	10/46	5645 ± 5675	3.5
Kosałka-Wegiel J et al., 2023, Poland (68)	S	24	31.3	14/10	64.5 ± 31.8	43	56	10/33	101.8 ± 82.4	5.33
Corrado A et al., 2024, Italy (51)	S	37	57.9	4/33	205.94 ± 124.75	57	58.9	5/52	679.85 ± 125.6	11.87

MDD, mean disease duration; M/F, male to female ratio; NR, not reported; P, plasma; S, serum; VEGF, vascular endothelial growth factor.

The risk of bias was considered low in 25 studies (28, 32, 47–51, 54–56, 58, 62, 63, 68–72, 74–79, 81) studies and moderate in the remaining 13 (22, 31, 33, 43, 52, 57, 60, 61, 66, 67, 73, 80, 82) (**Supplementary Table 2**).

**Figure 2:**
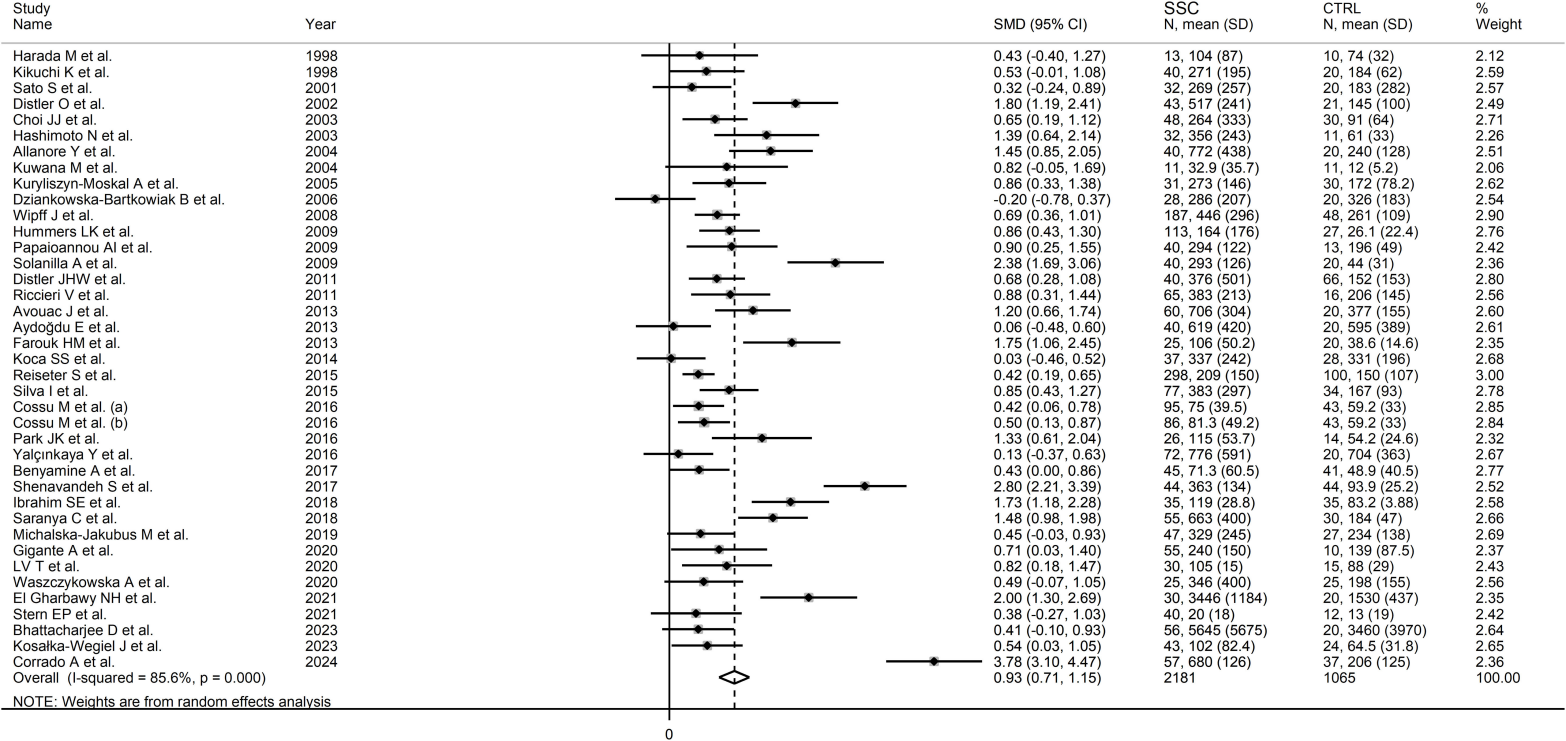
Forest plot of studies investigating VEGF concentrations in SSc patients and controls.

Pooled analyses showed that SSc patients had significantly higher VEGF concentrations than controls (SMD=0.93, 95% CI 0.71 to 1.15, p<0.001; I^2^ = 85.6%, p<0.001; **Figure 2**). Sensitivity analysis showed stability of the results, with pooled SMD values ranging between 0.67 and 0.96 (**Supplementary Figure 1**).

There was significant publication bias (Begg’s test, p=0.003; Egger’s test, p=0.005). The “trim-and-fill” method identified ten missing studies to be added to the left side of the funnel plot to ensure symmetry (**Supplementary Figure 2**). The resulting effect size was attenuated but still significant (SMD=0.56, 95% CI 0.30 to 0.82, p<0.001).

**Figure 3:**
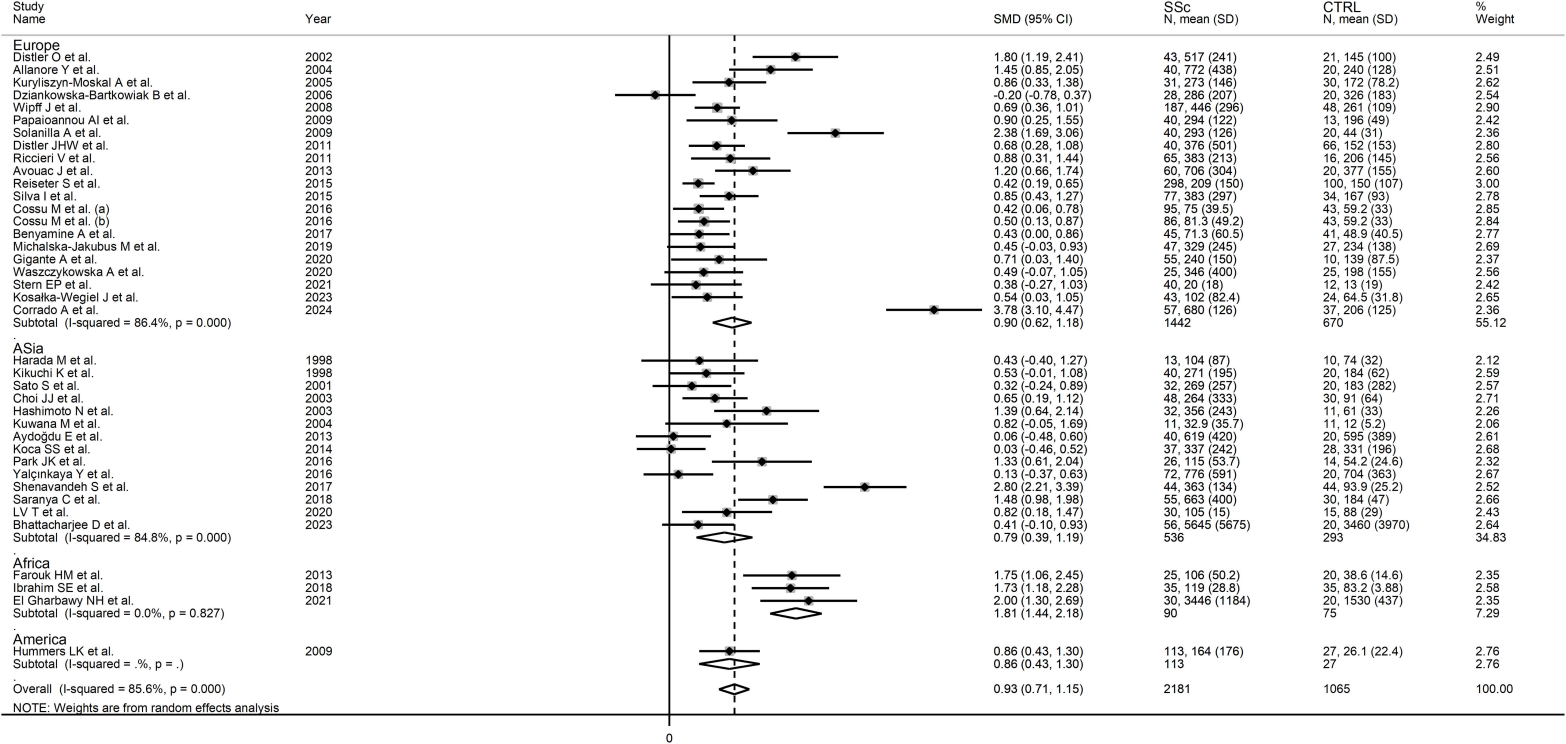
Forest plot of studies investigating VEGF concentrations in SSc patients and controls according to geographical area.

No significant associations were observed between the effect size and age (t=-1.02, p=0.31), male-to-female ratio (t=1.51, p=0.14), year of publication (t=0.91, p=0.37), number of participants (t=-0.70, p=0.49), mean SSc duration (t=0.60, p=0.55), or use of glucocorticoids (t=-1.85, p=0.08), immunosuppressors (t=-1.05, p=0.32), or vasodilators (t=-0.37, p=0.72) in univariate meta-regression analysis. In sub-group analysis, the pooled SMD of studies conducted in Africa (SMD=1.81, 95% CI 1.44 to 2.18, p<0.001; I^2^ = 0.0%, p=0.827) was significantly higher (p=0.039) than that of studies conducted in Asia (SMD=0.79, 95% CI 0.39 to 1.19, p<0.001; I^2^ = 84.8%, p<0.001) but not (p=0.08) Europe (SMD=0.90, 95% CI 0.62 to 1.18, p<0.001; I^2^ = 86.4%, p<0.001; **Figure 3**), with a virtually absent heterogeneity in the African subgroup. Non-significant differences (p=0.53) in pooled SMD were observed between studies measuring serum (SMD=0.89, 95% CI 0.65 to 1.13, p<0.001; I^2^ = 86.8%, p<0.001) and plasma (SMD=1.09, 95% CI 0.61 to 1.91, p=1.57; I^2^ = 79.1%, p<0.001). Finally, the pooled SMD was non-significantly different (p=0.12) between studies using an enzyme-linked immunosorbent assay (SMD=1.03, 95% CI 0.77 to 1.28, p<0.001; I^2^ = 87.4%, p<0.001) and a platform for multi-analyte profiling (SMD=0.49, 95% CI 0.31 to 0.67, p<0.001; I^2^ = 0.0%, p=0.52), with a virtually absent between-study variance in the multi-analyte profiling subgroup.

The overall level of certainty was upgraded to moderate (level 3) after considering the low-moderate risk of bias in all studies (no change), the high but partially explainable heterogeneity (no change), the lack of indirectness (no change), the large effect size (SMD=0.93; upgrade one level) (83), and the presence of publication bias which was addressed using the “trim-and-fill” method (no change).

### Localized vs. diffuse disease

Eleven studies investigated VEGF concentrations in 228 SSc patients with diffuse form and 279 with localized form (28, 31, 47, 52, 54, 57, 65, 66, 76, 78, 81) (**Table 2**). Five studies were conducted in Europe (28, 47, 52, 54, 81), five in Asia (31, 65, 66, 76, 78), and one in Africa (57). Enzyme-linked immunosorbent assay was used in all studies except one which used a platform for multi-analyte detection (52). Except for one study (57), measurements were conducted in serum.

**Table 2:** Summary of studies reporting VEGF concentrations in SSc patients with localized and diffuse disease.

	Localized		Diffuse	
Study	n	VEGF (Mean ± SD)	n	VEGF (Mean ± SD)
Kikuchi K et al., 1998, Japan (66)	20	183 ± 89	20	360 ± 233
Distler O et al., 2002, Italy (28)	20	380 ± 183	23	532 ± 274
Choi JJ et al., 2003, Korea (31)	27	125 ± 169	21	440 ± 306
Allanore Y et al., 2004, France (47)	23	690 ± 406	17	813 ± 497
Distler JHW et al., 2011, Switzerland (54)	20	336 ± 438	20	416 ± 563
Farouk HM et al., 2013, Egypt (57)	15	110 ± 12	10	108 ± 21
Cossu M et al., 2016, Italy (52)	51	84.6 ± 50.2	36	76.54 ± 48.04
Shenavandeh S et al., 2017, Iran (78)	17	181.6 ± 310	27	514.4 ± 1167
Kawashiri S et al., 2018, Japan (65)	44	379 ± 317	16	343 ± 167
Saranya C et al., 2018, India (76)	25	618 ± 413	30	682 ± 390
Waszczykowska A et al., 2020, Poland (81)	17	384.76 ± 467.19	8	296.3 ± 218.3

VEGF, vascular endothelial growth factor.

The risk of bias was considered low in seven studies (28, 47, 54, 65, 76, 78, 81) and moderate in the remaining four (31, 52, 57, 66) (**Supplementary Table 2**).

**Figure 4:**
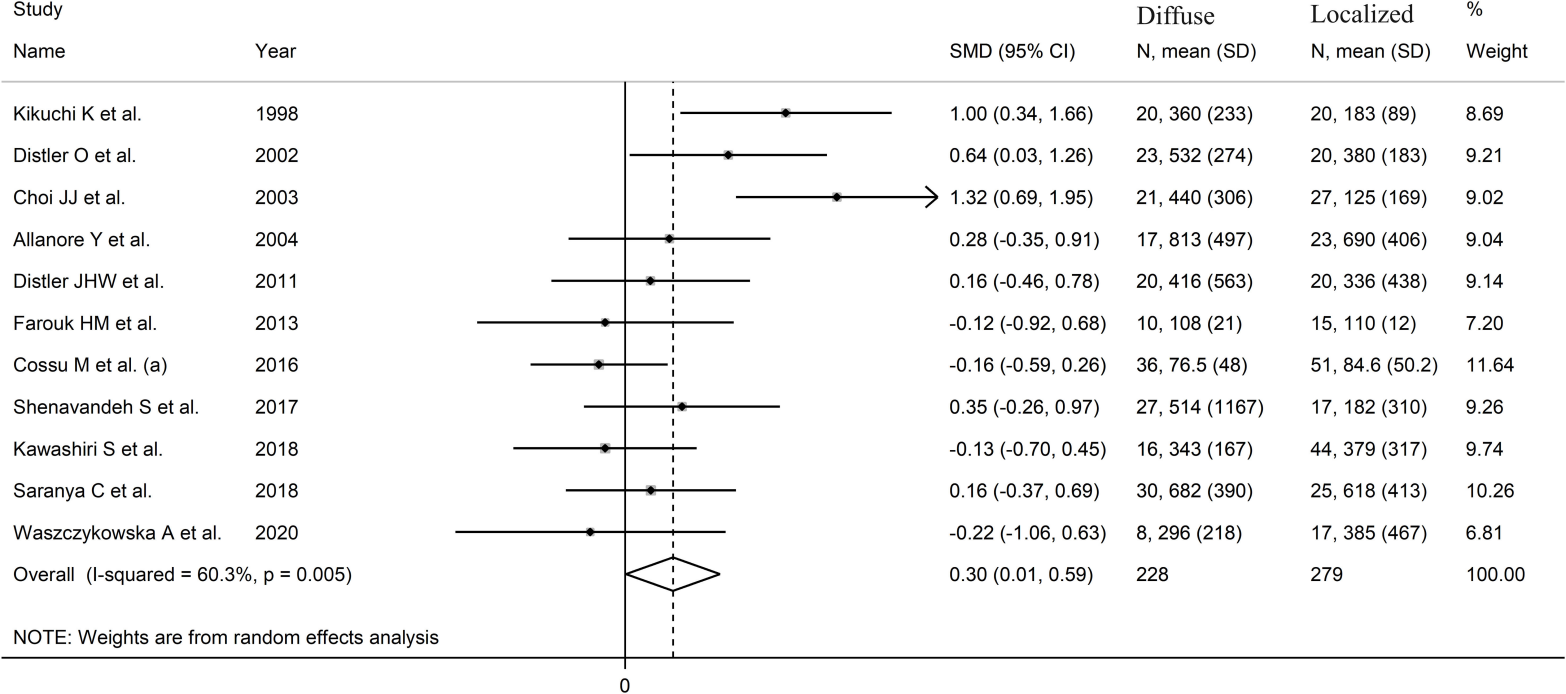
Forest plot of studies investigating VEGF concentrations in SSc patients with diffuse or localized form.

The pooled analysis showed that SSc patients with diffuse disease had significantly higher VEGF concentrations than those with localized disease (SMD=0.30, 95% CI 0.01 to 0.59, p=0.046; I^2^ = 60.3%, p=0.005; **Figure 4**). The results were stable in sensitivity analysis, with pooled SMD values ranging between 0.19 and 0.36 (**Supplementary Figure 3**).

There was no publication bias (Begg’s test, p=0.35; Egger’s test, p=0.46). Accordingly, the “trim-and-fill” method did not identify any missing study to be added to the funnel plot to ensure symmetry (**Supplementary Figure 4**). The resulting effect size was increased and still significant (SMD=0.51, 95% CI 0.08 to 0.94; p=0.021).

**Figure 5:**
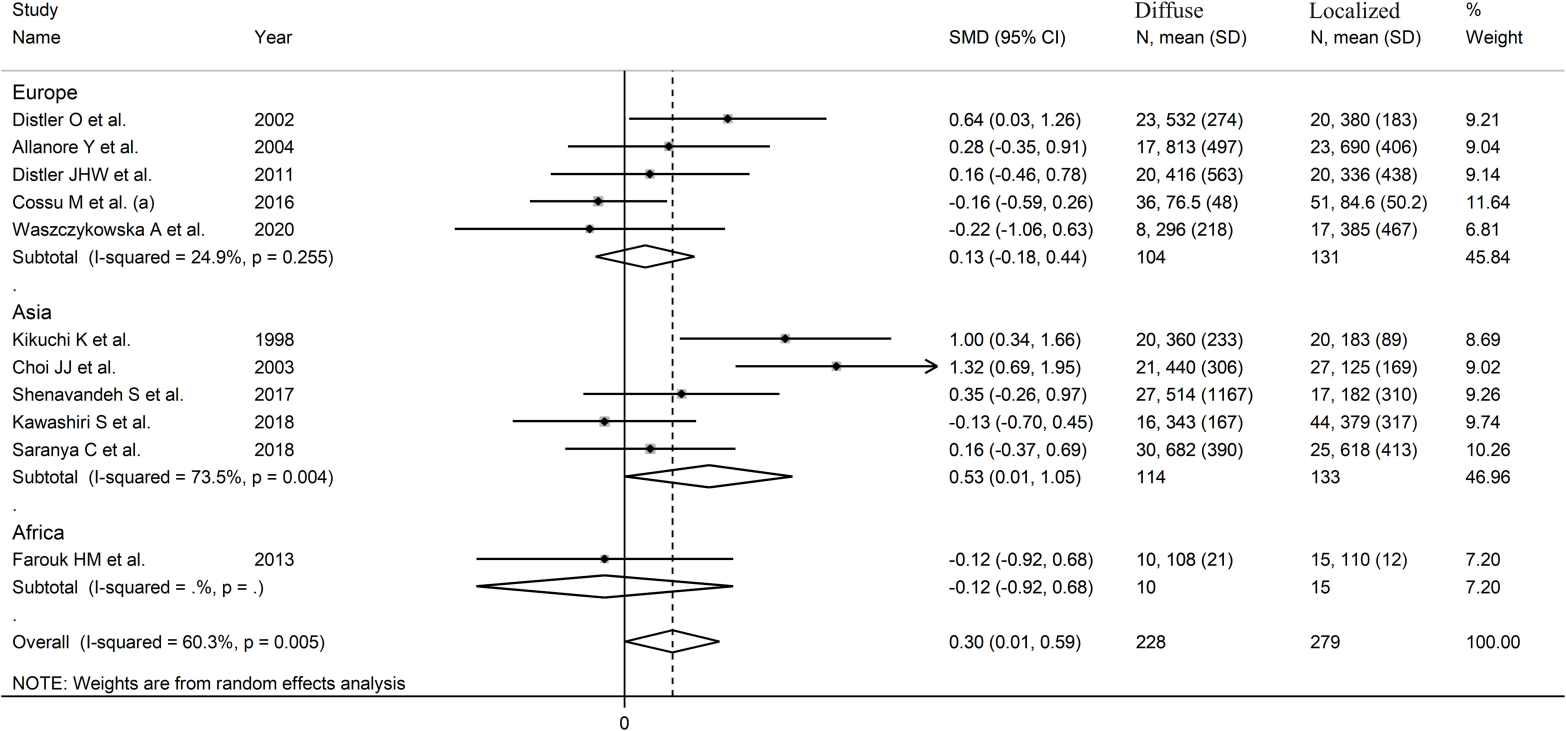
Forest plot of studies investigating VEGF concentrations in SSc patients with localized or diffuse form according to geographical area where the study was conducted.

A limited number of meta-regression and subgroup analyses could be performed due to the limited number of studies. No significant associations were found between the effect size and sample size (t=-0.65, p=0.53). By contrast, there was a significant correlation with the year of publication (t=-3.95, p=0.003; **Supplementary Figure 5A**), as also confirmed by cumulative analysis performed using the metacum command (**Supplementary Figure 5B**). In sub-group analysis, the pooled SMD was significantly different in studies conducted in Asia (SMD=0.53, 95% CI 0.01 to 1.05, p=0.048; I^2^ = 73.5%, p=0.004) but not Europe (SMD=0.13, 95% CI -0.18 to 0.44, p=0.41; I^2^ = 24.9%, p=0.25; **Figure 5**), with a substantial reduction in heterogeneity in the European subgroup.

The overall level of certainty remained low (level 2) after considering the low-moderate risk of bias in all studies (no change), the high but partially explainable heterogeneity (no change), the lack of indirectness (no change), the small effect size (SMD=0.30; no change) (83), and the lack of publication bias (no change).

### Capillaroscopy pattern

Five studies reported serum VEGF concentrations in SSc patients stratified according to the capillaroscopy pattern (28, 32, 59, 71, 82) (**Table 3**). Four studies were conducted in Europe (28, 32, 59, 71) and one in Asia (82). All studies used an enzyme-linked immunosorbent assay except one, which used used a platform for multi-analyte detection (82).

**Table 3:** Summary of studies reporting VEGF concentrations in SSc patients according to capillaroscopy pattern.

	Early		Active		Late	
Study	n	VEGF (Mean ± SD)	n	VEGF (Mean ± SD)	n	VEGF (Mean ± SD)
Distler O et al., 2002, Italy (28)	6	427 ± 218	22	465 ± 275	14	602 ± 291
Avouac J et al., 2013, France (32)	44	556 ± 198	22	572 ± 259	24	845 ± 353
Yalçınkaya Y et al., 2016, Turkey (82)	10	996 ± 904	37	745 ± 570	25	733 ± 464
Gigante A et al., 2017, Italy (59)	22	274.4 ± 259.7	35	268 ± 221.5	34	305 ± 278
Michalska-Jakubus M et al., 2019, Poland (71)	14	294.85 ± 237.76	14	252.68 ± 234.59	19	411.48 ± 245.78

VEGF, vascular endothelial growth factor.

**Figure 6:**
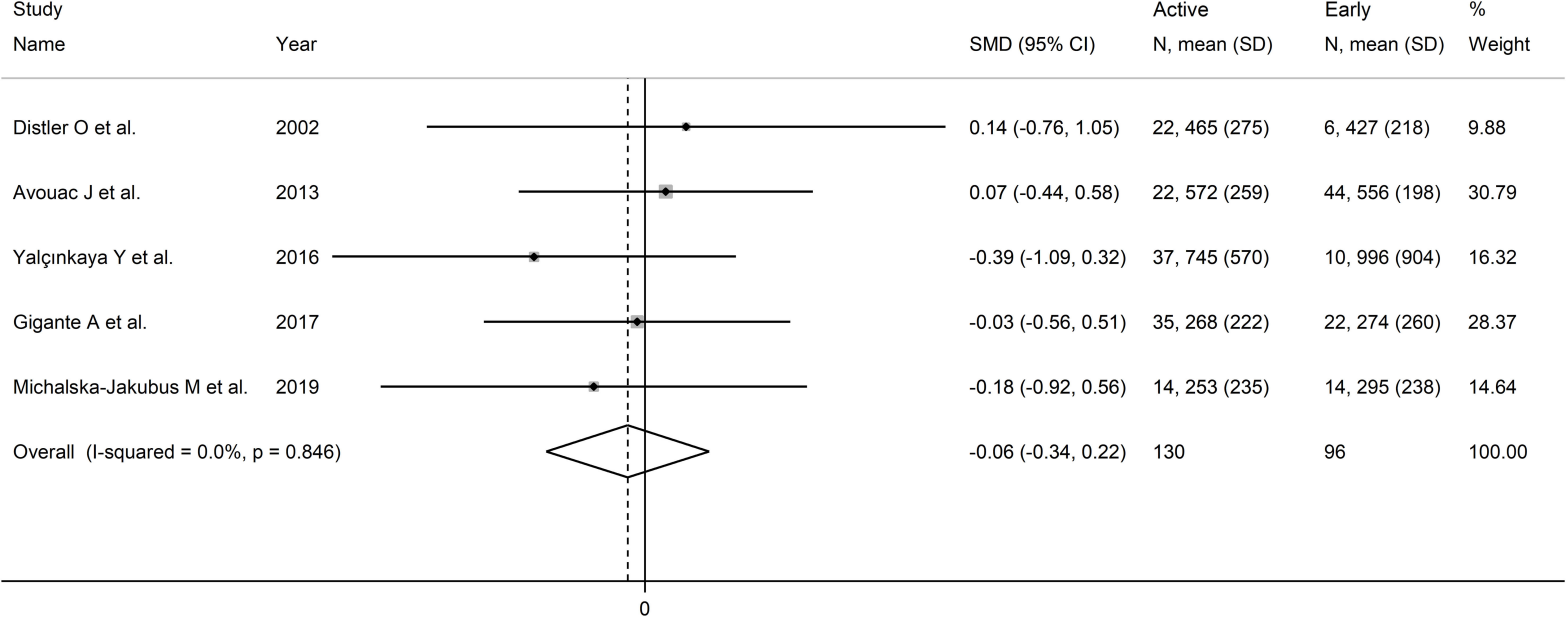
Forest plot of studies investigating VEGF concentrations in SSc patients according to capillaroscopy pattern (early vs. active).

Pooled analysis showed non-significant differences in VEGF concentrations between early and active SSc patients (SMD=-0.06, 95% CI -0.34 to 0.22, p=0.68; I^2^ = 0.0%, p=0.85; **Figure 6**). Sensitivity analysis confirmed the stability of the results, with pooled SMD values ranging between -0.12 and 0.00 (**Supplementary Figure 6**). Assessment of publication bias, meta-regression and sub-group analyses could not be performed because of the small number of studies.

**Figure 7:**
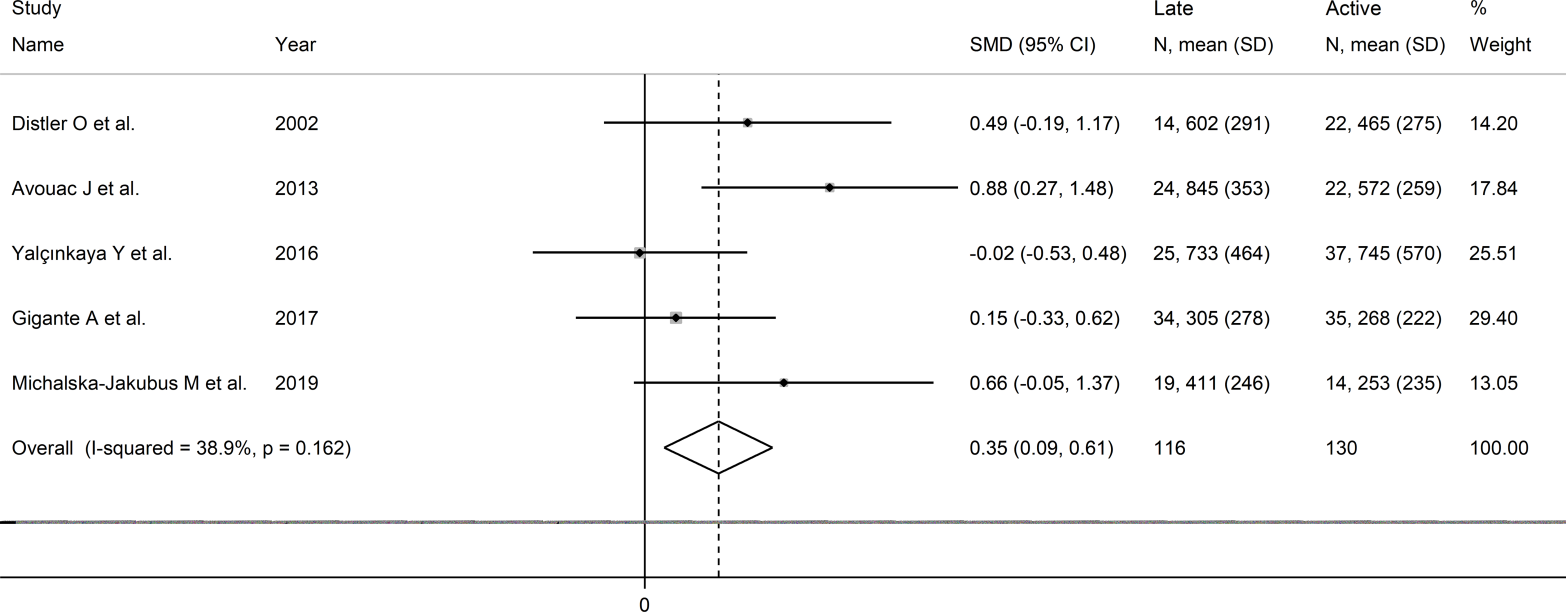
Forest plot of studies investigating VEGF concentrations according to capillaroscopy pattern (active vs. late).

Pooled analysis showed that VEGF concentrations was higher in late than active SSc patients (SMD=0.35, 95% CI 0.09 to 0.61, p=0.008; I^2^ = 38.9%, p=0.16; **Figure 7**). Sensitivity analysis confirmed stability of the results, with pooled SMD values ranging between 0.23 and 0.49 (**Supplementary Figure 7**). Assessment of publication bias, meta-regression and sub-group analyses could not be performed because of the small number of studies.

**Figure 8:**
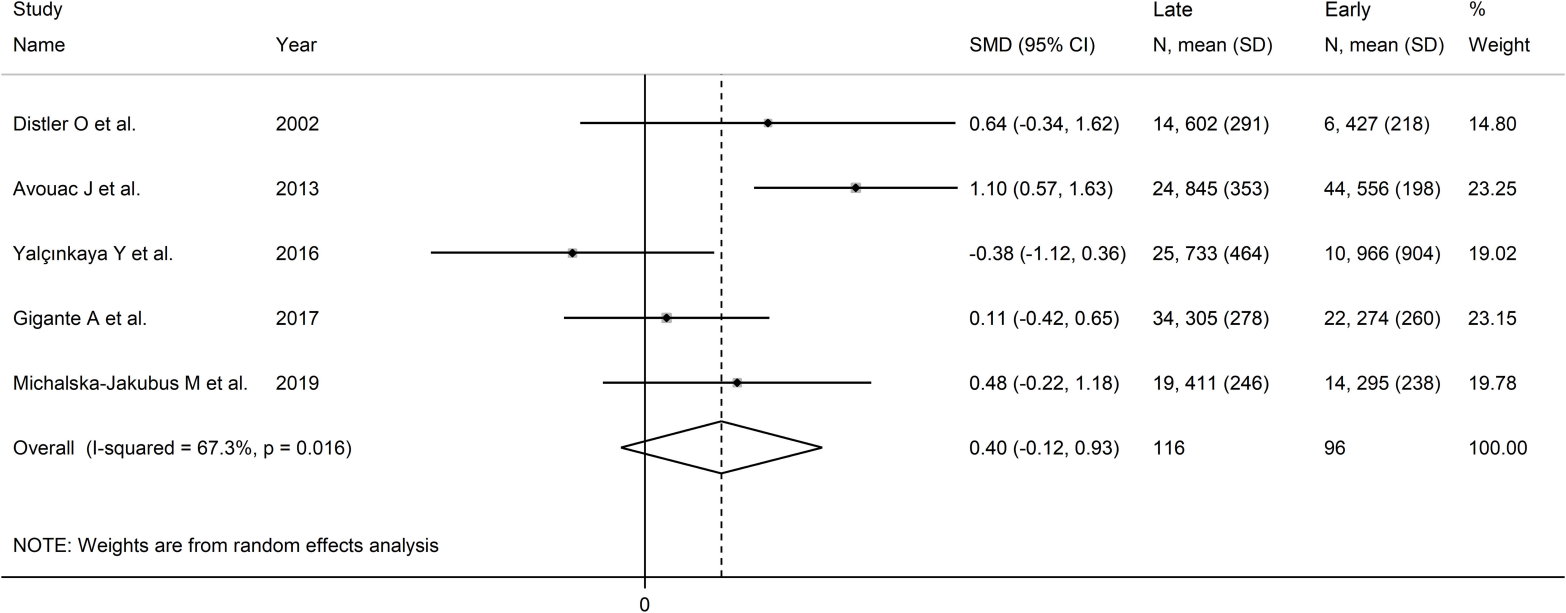
Forest plot of studies investigating VEGF concentrations according to capillaroscopy pattern (early vs. late).

Pooled results showed that VEGF concentrations were non-significantly different between late and early SSc patients (SMD=0.40, 95% CI -0.13 to 0.93, p=0.14; I^2^ = 67.3%, p=0.016; **Figure 8**). The results were stable in sensitivity analysis (pooled SMD values ranged between 0.17 and 0.58; **Supplementary Figure 8**). Assessment of publication bias, meta-regression and sub-group analyses could not be performed because of the small number of studies.

The overall level of certainty was downgraded to very low (level 1) because of the lack of assessment of publication bias.

### Digital ulcers

Seven studies investigated serum VEGF concentrations in 562 SSc patients, 265 without and 297 with digital ulcers (28, 52, 58, 59, 65, 79) (**Table 4**). All studies were conducted in Europe except one which was conducted in Asia (65). All studies used an enzyme-linked immunosorbent assay except one, which used used a platform for multi-analyte detection (52).

**Table 4:** Summary of studies reporting VEGF concentrations in SSc patients with and without complications.

Study	Absence of complication		Presence of complication	
	n	VEGF (Mean ± SD)	n	VEGF (Mean ± SD)
Digital Ulcers				
Distler O et al., 2002, Italy (28)	27	541 ± 242	16	352 ± 187
Silva I et al., 2015, Portugal (79)	39	513 ± 445	38	250 ± 145
Cossu M et al., 2016, Italy (52)	35	71.28 ± 47.8	144	79.6 ± 43.36
Gigante A et al., 2017, Italy (59)	51	259 ± 259.2	40	302 ± 244.8
Kawashiri S et al., 2018, Japan (65)	50	367 ± 303	10	453 ± 173
Gigante A et al., 2020, Italy (58)	36	249 ± 80	19	226 ± 76
Corrado A et al., 2024, Italy (51)	27	572.53 ± 63.91	30	764.22 ± 94.24
Interstitial lung disease				
Dziankowska-Bartkowiak B et al., 2006, Poland (55)	8	158 ± 112	20	276 ± 223
Wipff J et al., 2008, France (22)	100	393.8 ± 268.2	87	488.2 ± 322.4
Cossu M et al., 2016, Italy (52)	33	85.17 ± 46.3	146	76.35 ± 43.75
Kawashiri S et al., 2018, Japan (65)	36	354 ± 317	24	390 ± 251
Saranya C et al., 2018, India (76)	34	508 ± 358	21	821 ± 324
Pulmonary artery hypertension				
Wipff J et al., 2008, France (22)	170	430.2 ± 195.3	17	579.3 ± 270
Papaioannou AI et al., 2009, Greece (72)	20	239 ± 59	20	360 ± 156
Jouvray M et al., 2018, France (64)	94	384.1 ± 238.9	12	445.3 ± 176
Corrado A et al., 2024, Italy (51)	50	638.02 ± 104.5	7	844.14 ± 112.23
Telangiectasias				
Solanilla A et al., 2009, France (80)	16	201 ± 100	14	475 ± 201
Cossu M et al., 2016, Italy (52)	92	74.84 ± 43.17	87	81.29 ± 45.36
Michalska-Jakubus M et al., 2019, Poland (71)	30	168.19 ± 142.24	17	377 ± 250

**Figure 9:**
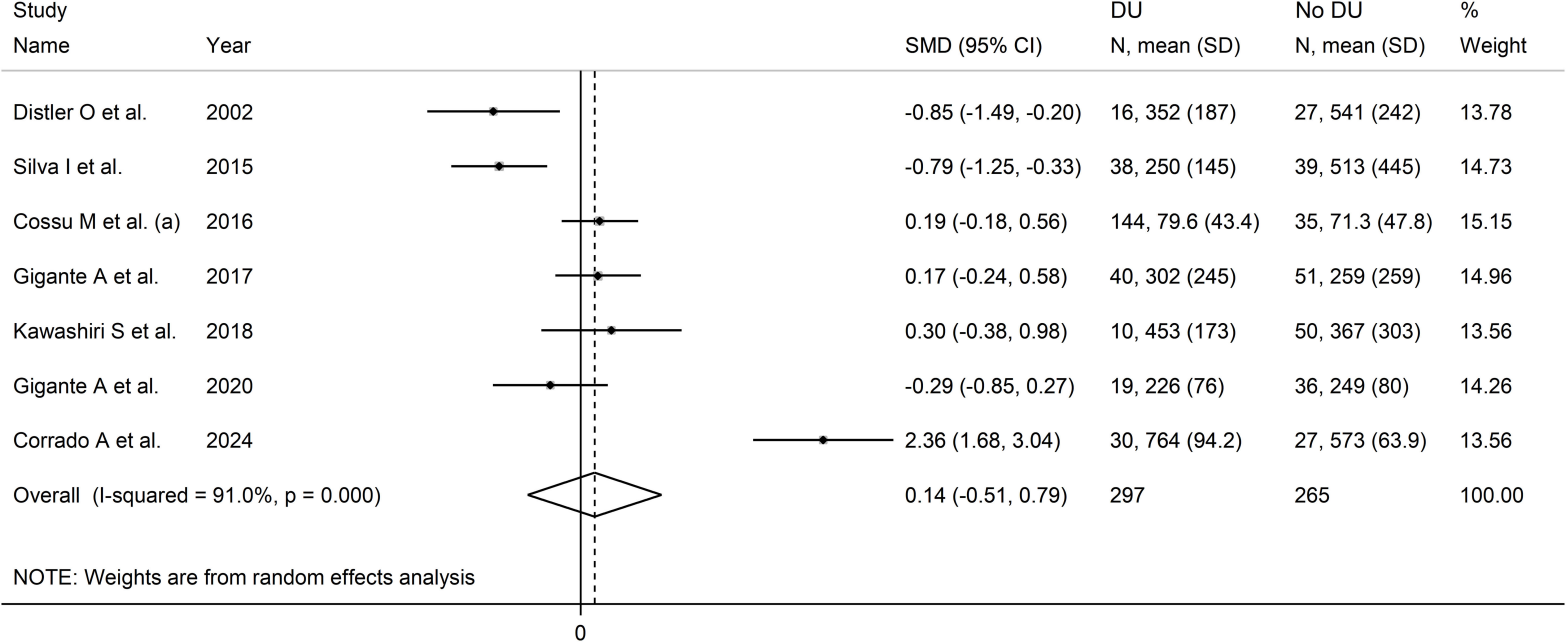
Forest plot of studies investigating VEGF concentrations in SSc patients with or without digital ulcers.

Pooled results showed non-significant between-group differences in VEGF concentrations (SMD=0.14, 95% CI -0.51 to 0.79, p=0.67; I^2^ = 91.0%, p<0.001; **Figure 9**). Sensitivity analysis showed stability of the results, with an effect size ranging between -0.20 and 0.30 (**Supplementary Figure 9**).

Assessment of publication bias, meta-regression and sub-group analyses could not be performed because of the small number of studies. Consequently, the overall certainty of evidence was downgraded to very low (level 1).

### Interstitial lung disease

Five studies investigated serum VEGF concentrations in 509 SSc patients, 211 without and 298 with interstitial lung disease (22, 52, 55, 65, 76) (**Table 4**). Three studies were performed in Europe (22, 52, 55) and two in Asia (65, 76). All studies used an enzyme-linked immunosorbent assay except one, which used used a platform for multi-analyte detection (52).

**Figure 10:**
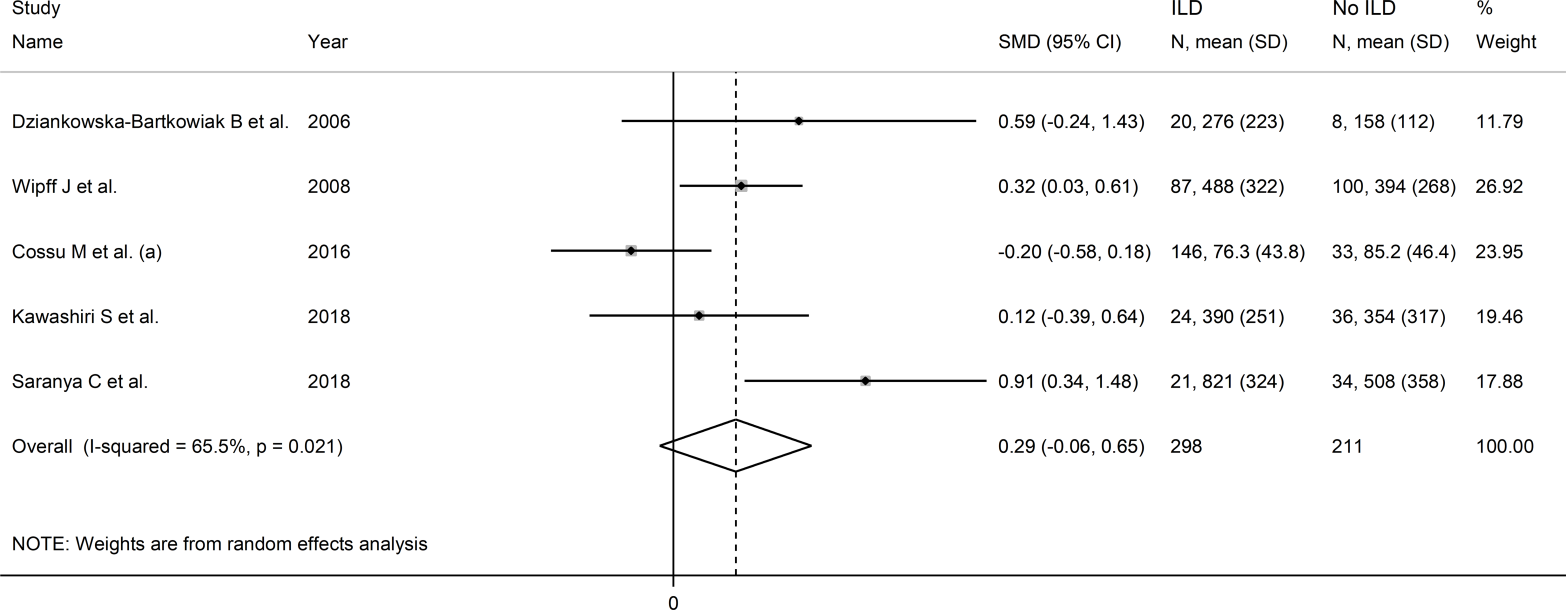
Forest plot of studies investigating VEGF concentrations in SSc patients with or without interstitial lung disease.

Pooled results showed that SSc patients with interstitial lung disease had non-significantly higher VEGF concentrations than SSc patients without (SMD=0.29, 95% CI -0.06 to 0.65, p=0.11; I^2^ = 65.5%, p=0.021; **Figure 10**). Sensitivity analysis showed that the pooled SMD value become significant after removing the study by Cossu et al. (52) (SMD=0.43, 95% CI 0.12 to 0.74, p=0.001, I^2^ = 34.9%, p=0.23), with a concomitant reduction in between-study variance (**Supplementary Figure 10**).

Assessment of publication bias, meta-regression and sub-group analyses could not be performed because of the small number of studies. Consequently, the overall certainty of evidence was downgraded to very low (level 1).

### Pulmonary hypertension

**Figure 11:**
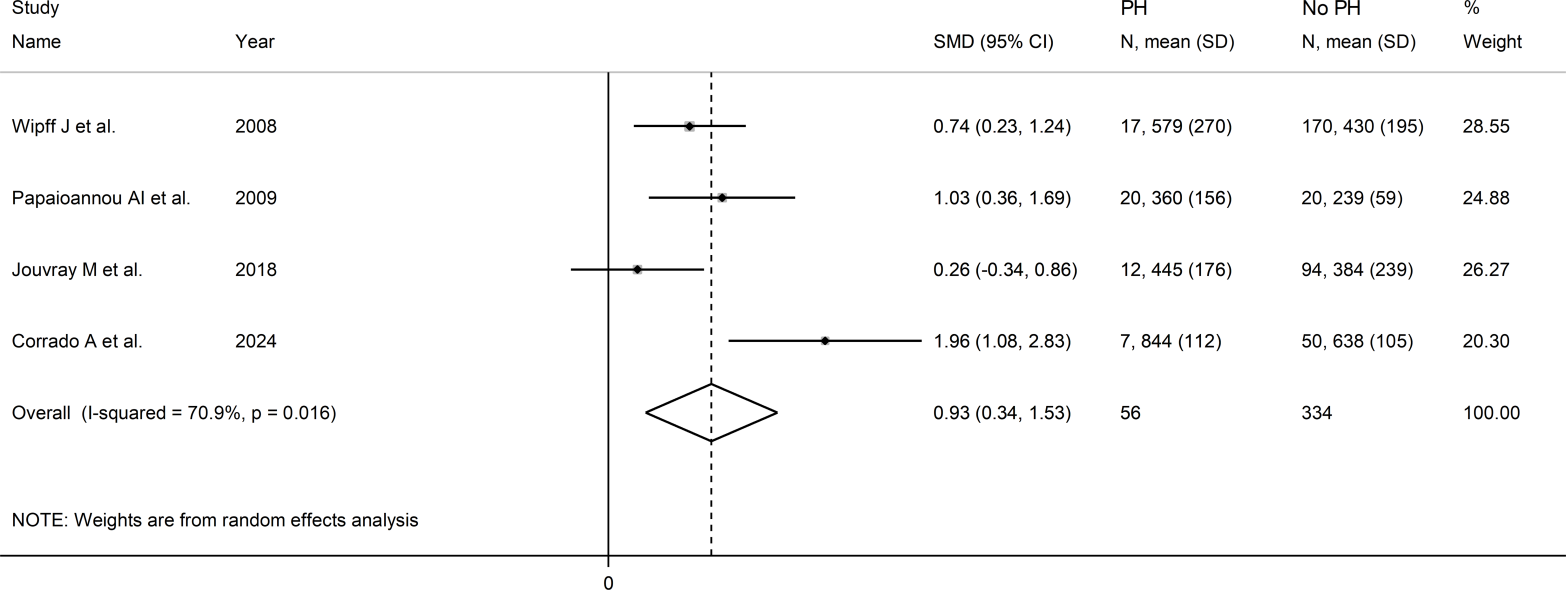
Forest plot of studies investigating VEGF concentrations in SSc patients with and without pulmonary hypertension.

Four studies investigated serum VEGF concentrations in 390 SSc patients, 334 without and 56 with pulmonary hypertension (22, 51, 64, 72). All studies were conducted in Europe and used an enzyme-linked immunosorbent assay. Pooled results showed that SSc patients with pulmonary hypertension had significantly higher VEGF concentrations than SSc patients without (SMD=0.93, 95% CI 0.34 to 1.53, p=0.002; I^2^ = 70.9%, p=0.016; **Figure 11**).

Assessment of sensitivity, publication bias, meta-regression and sub-group analyses could not be performed because of the small number of studies. Consequently, the overall certainty of evidence was downgraded to very low (level 1).

### Telangiectasias

Three studies investigated VEGF concentrations in 256 SSc patients, 138 without and 118 with telangiectasias (52, 71, 80). All studies were conducted in Europe and used an enzyme-linked immunosorbent assay. Two studies measured serum (52, 71) and the remaining one plasma (80).

**Figure 12:**
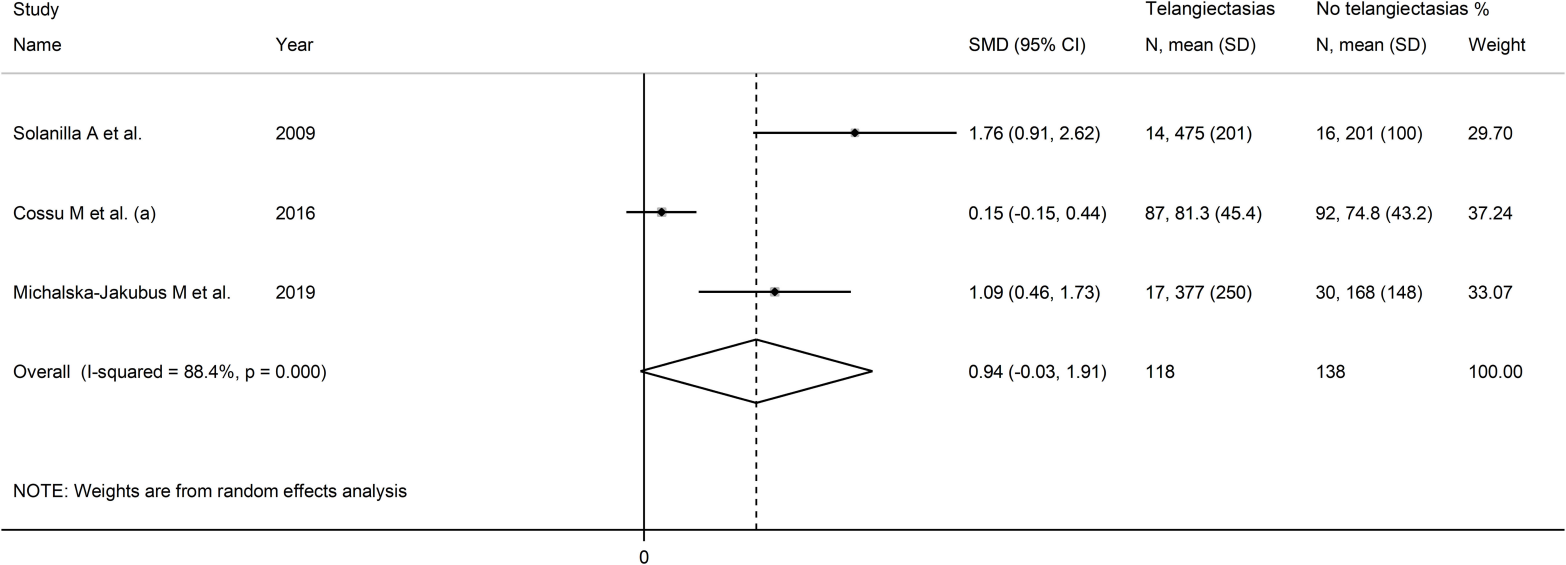
Forest plot of studies investigating VEGF concentrations in SSc patients with and without telangiectasias.

Pooled results showed a non-significant trend toward higher VEGF concentrations in patients with telangiectasias (SMD=0.94, 95% CI -0.03 to 1.91, p=0.058, I^2^ = 88.4%, p<0.001; **Figure 12**).

Assessment of sensitivity, publication bias, meta-regression and sub-group analyses could not be performed because of the small number of studies. Consequently, the overall certainty of evidence was downgraded to very low (level 1).

### Alveolitis

One study performed in Italy reported VEGF concentrations in 55 SSc patients, 27 without and 28 with alveolitis (53). Patients with alveolitis had non-significantly higher VEGF concentrations compared to those without (median: 53.9 pg/mL, IQR 5.5–184.3 pg/mL vs. 31.8 pg/mL, IQR 5.5–321.8 pg/mL, p>0.05).

## Discussion

This systematic review and meta-analysis has highlighted the presence of significant elevations in plasma or serum VEGF concentrations in patients with SSc when compared to healthy controls. In further analyses specifically in SSc patients, higher VEGF concentrations were significantly associated with diffuse disease, late vs. active video capillaroscopy pattern, and pulmonary hypertension. The alterations in VEGF concentrations associated with microvascular (video capillaroscopy pattern) and macrovascular (pulmonary hypertension) complications are also likely to reflect a state of nitric oxide dysregulation and endothelial dysfunction (84–87). By contrast, there were no significant associations with other complications such as digital ulcers, interstitial lung disease, or telangiectasias, whereas only one study reported non-significant differences in VEGF concentrations between SSc patients with and without alveolitis.

Meta-regression and subgroup analysis of studies investigating VEGF concentrations in SSc patients and controls showed non-significant associations between the effect size of the reported differences and various patient and study characteristics, particularly mean SSc duration and use of established, e.g., immunosuppressors and vasodilators (88), and less common, e.g. corticosteroids (89), treatments. By contrast, significant associations were observed with the geographical location where the study was conducted with a significantly higher effect size in African than Asian, but not European, studies. Meta-regression and subgroup analyses of studies investigating VEGF in SSc patients with localized and diffuse disease showed a significant and inverse association between the effect size and publication year and the lack of significant differences in European studies when compared to studies conducted in Asia which reported significant differences. The lack of significant associations between the effect size of between-group differences in VEGF concentrations and mean disease duration suggests that VEGF concentrations are already increased during the early stages of SSc compared to the general population. However, such concentrations can further increase in SSc patients with more advanced disease, as suggested by the higher VEGF concentrations observed in SSc patients with late compared to active videocapillaroscopy pattern. Taken together, the results of this systematic review and meta-analysis suggest that measuring VEGF concentrations can be useful in assessing and managing patients with SSc during different stages of the disease. However, the role of VEGF in different clinical manifestations of SSc requires confirmation in further studies. Furthermore, prospective studies are warranted to determine whether VEGF may be useful not only as a diagnostic but also as a prognostic biomarker in SSc.

Studies conducted in experimental models of SSc using VEGF transgenic mice have shown that VEGF exerts dose-dependent pro-fibrotic effects (21). Notably, these effects were accompanied by ineffective angiogenesis and vasculopathy, a common feature in SSc patients (29). Therefore, alterations in VEGF are likely to reflect a common pathway involved in the development of vasculopathy, inefficient angiogenesis, and fibrosis in SSc (30). Notably, VEGF pre-mRNA can lead to the synthesis of two heterodimers exerting opposite effects on angiogenesis, VEGF165 (pro-angiogenic) and VEGF165b (anti-angiogenic) (90, 91). The relative overexpression of VEGF165b in SSc has been shown to be associated with increased expression of transforming growth factor-β1 and serine/arginine protein 55 splicing factor, exerting pro-fibrotic effects, in endothelial cells, keratinocytes, and fibroblasts as well as significant capillary morphological alterations (92). In our analyses, increased VEGF concentrations were particularly evident in SSc patients with diffuse disease, pulmonary hypertension, and late vs. active capillaroscopy pattern. Future studies should investigate whether VEGF165 and VEGF165b play a pathophysiological role in these subgroups as well as the therapeutic role of VEGF modulators (93, 94). Clearly, the identification of possible interventions targeting VEGF requires additional research to determine the most promising target(s), i.e., VEGF, VEGF165, or VEGF165b. Additional research should also investigate the possible influence of ethnicity and genetic factors in the complex interplay between VEGF and SSc, as also suggested in our subgroup analyses (95).

Our study has several strengths, include the assessment of VEGF concentrations in a wide range of SSc subtypes (extent of fibrosis, video capillaroscopy patterns, and key clinical complications), the evaluation of the certainty of evidence for each endpoint, and the evaluation of specific study and patient characteristics associated with the effect size. One important limitation is the high heterogeneity observed. However, this could be partially explained in our sub-group analyses (presence of SSc: study location and analytical method used; disease type: study location). Another limitation is represented by the limited number of studies providing details regarding the presence of disease states and/or risk factors associated per se with alterations in circulating VEGF concentrations (96–98).

In conclusion, our study has shown significant elevations in VEGF concentrations in SSc and, particularly, diffuse disease, specific video capillaroscopy patterns, and pulmonary hypertension. Pending further prospective studies investigating a wide range of SSc subtypes in different geographical locations, measuring VEGF concentrations might assist in assessing and managing patients with this chronic and disabling autoimmune disorder.

## Data Availability

The raw data supporting the conclusions of this article will be made available by the authors, without undue reservation.
