# Supplementary figures and images for: Vascular endothelial growth factor as a potential biomarker in systemic sclerosis: a systematic review and meta-analysis
Source: Front Immunol. 2024 Nov 28;15:1442913. doi: 10.3389/fimmu.2024.1442913 (PMC11634811; doi:10.3389/fimmu.2024.1442913)

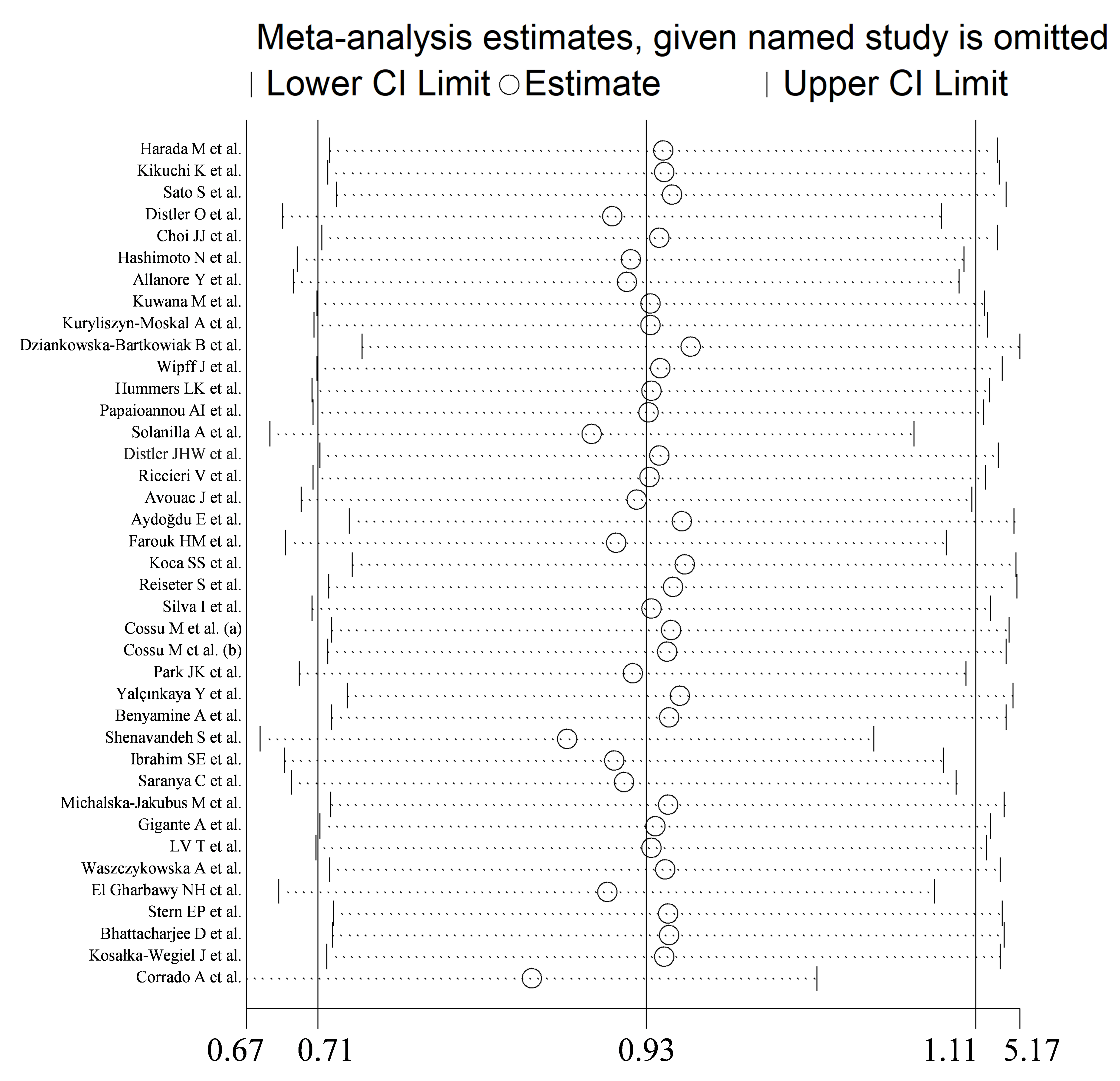

Supplement: Supplementary file 1 [file Image1.tif]

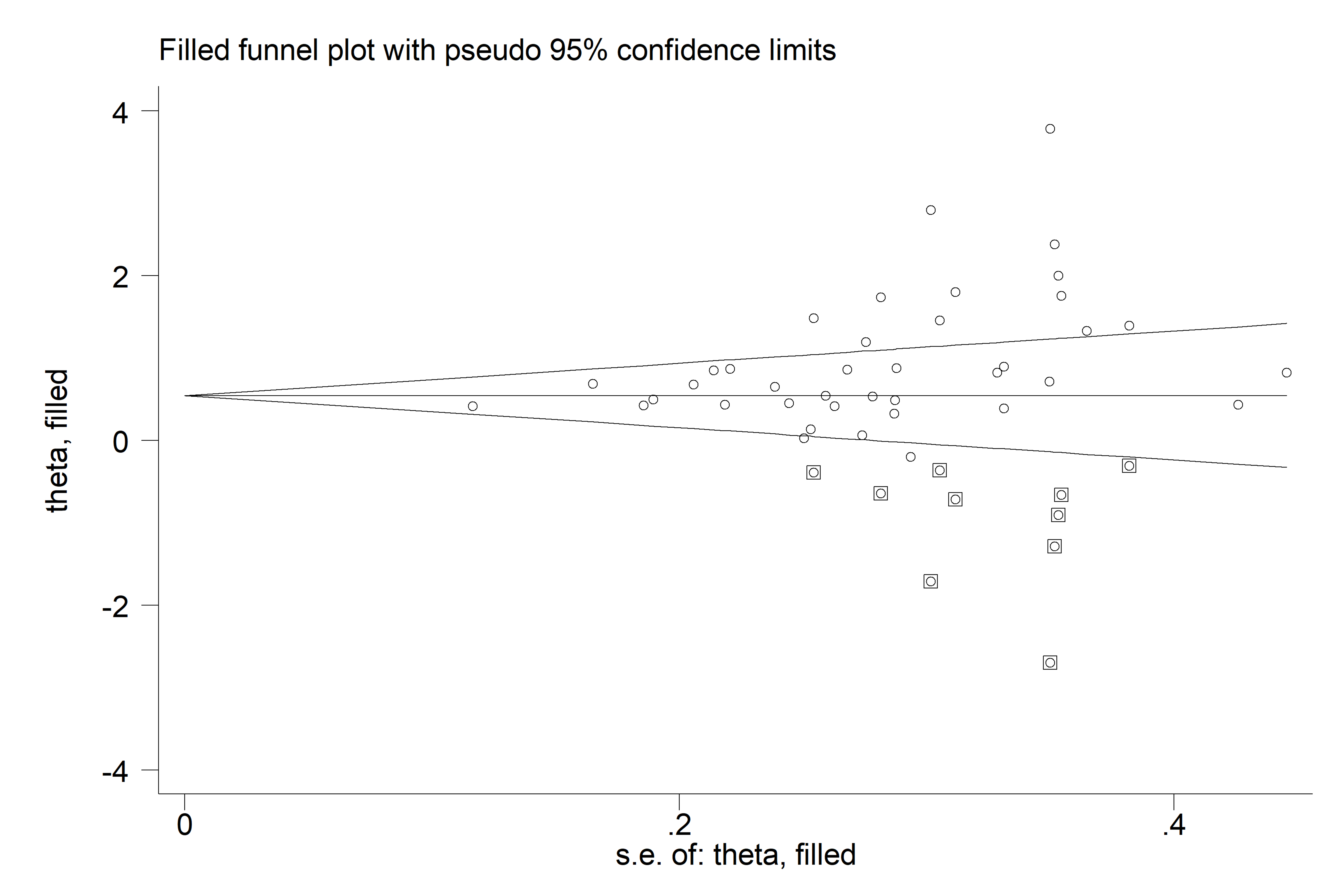

Supplement: Supplementary file 2 [file Image2.tif]

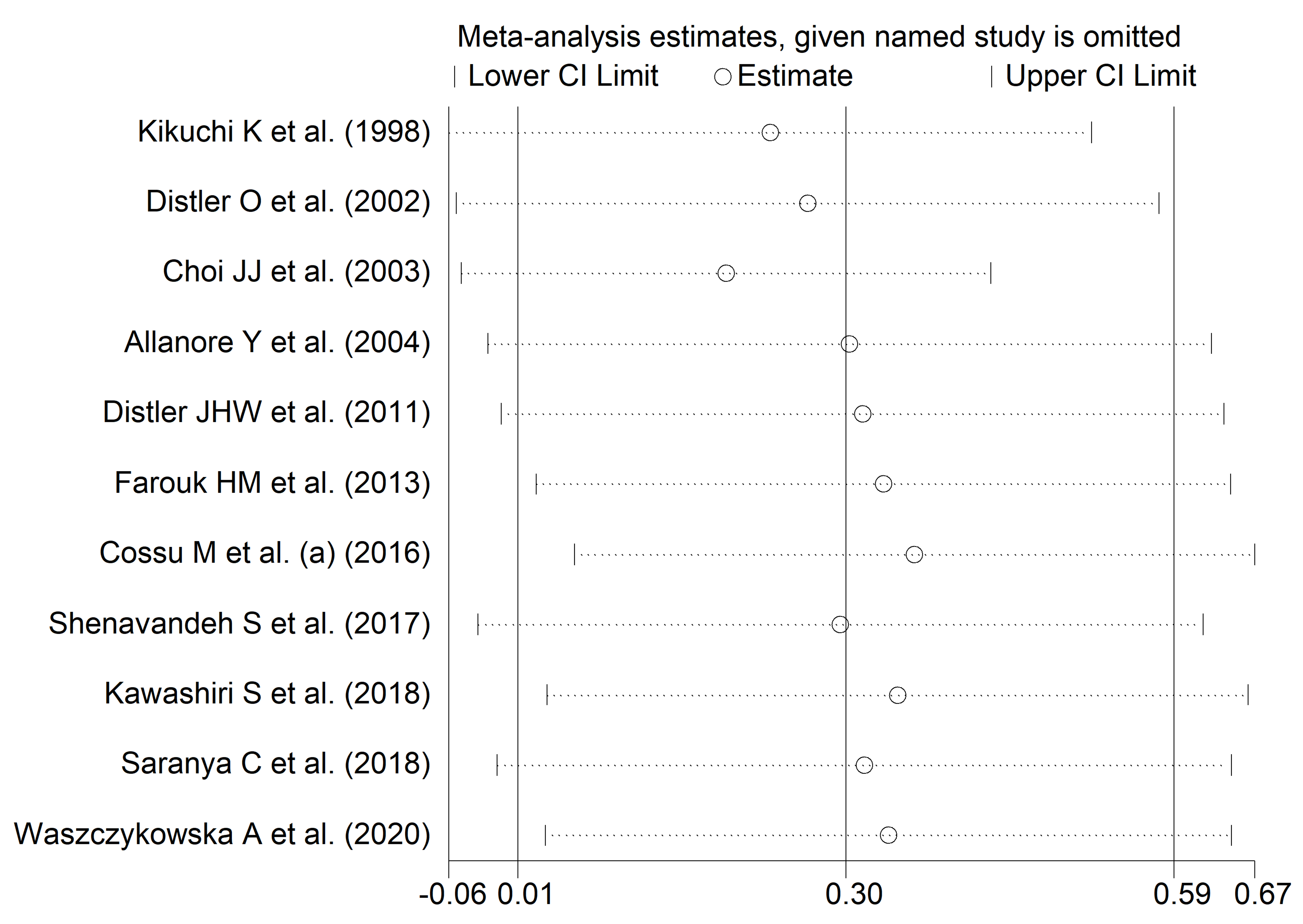

Supplement: Supplementary file 3 [file Image3.tif]

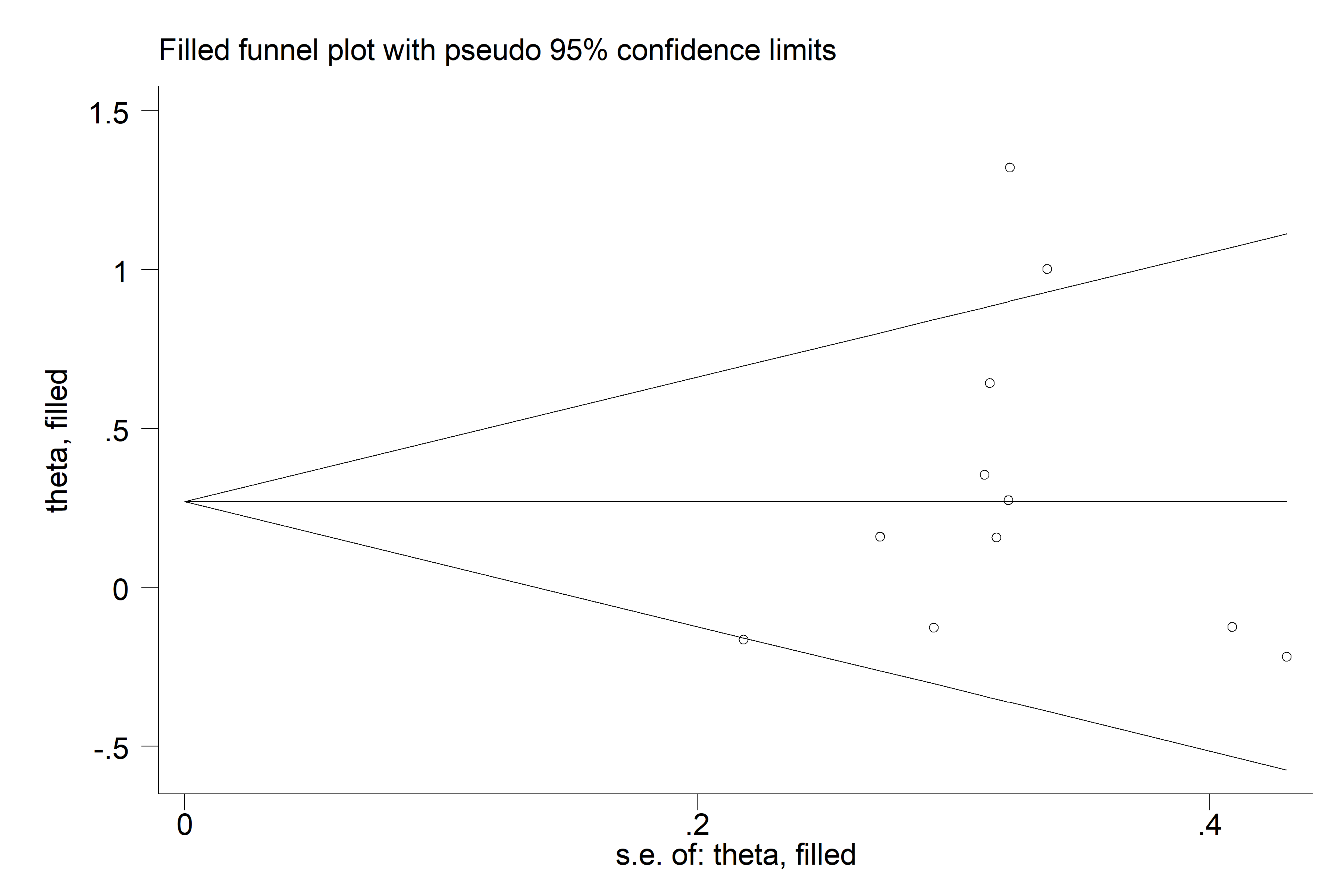

Supplement: Supplementary file 4 [file Image4.tif]

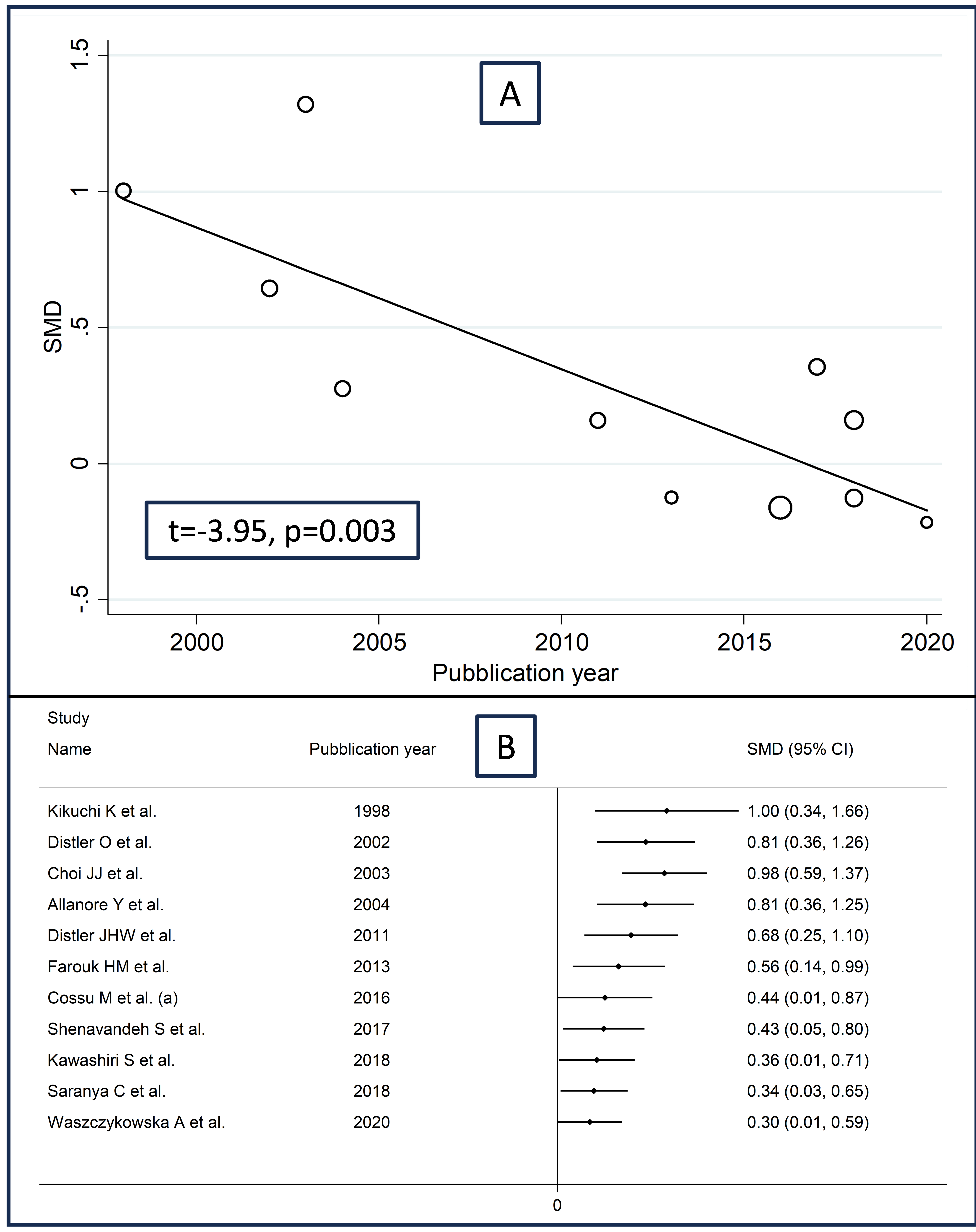

Supplement: Supplementary file 5 [file Image5.tif]

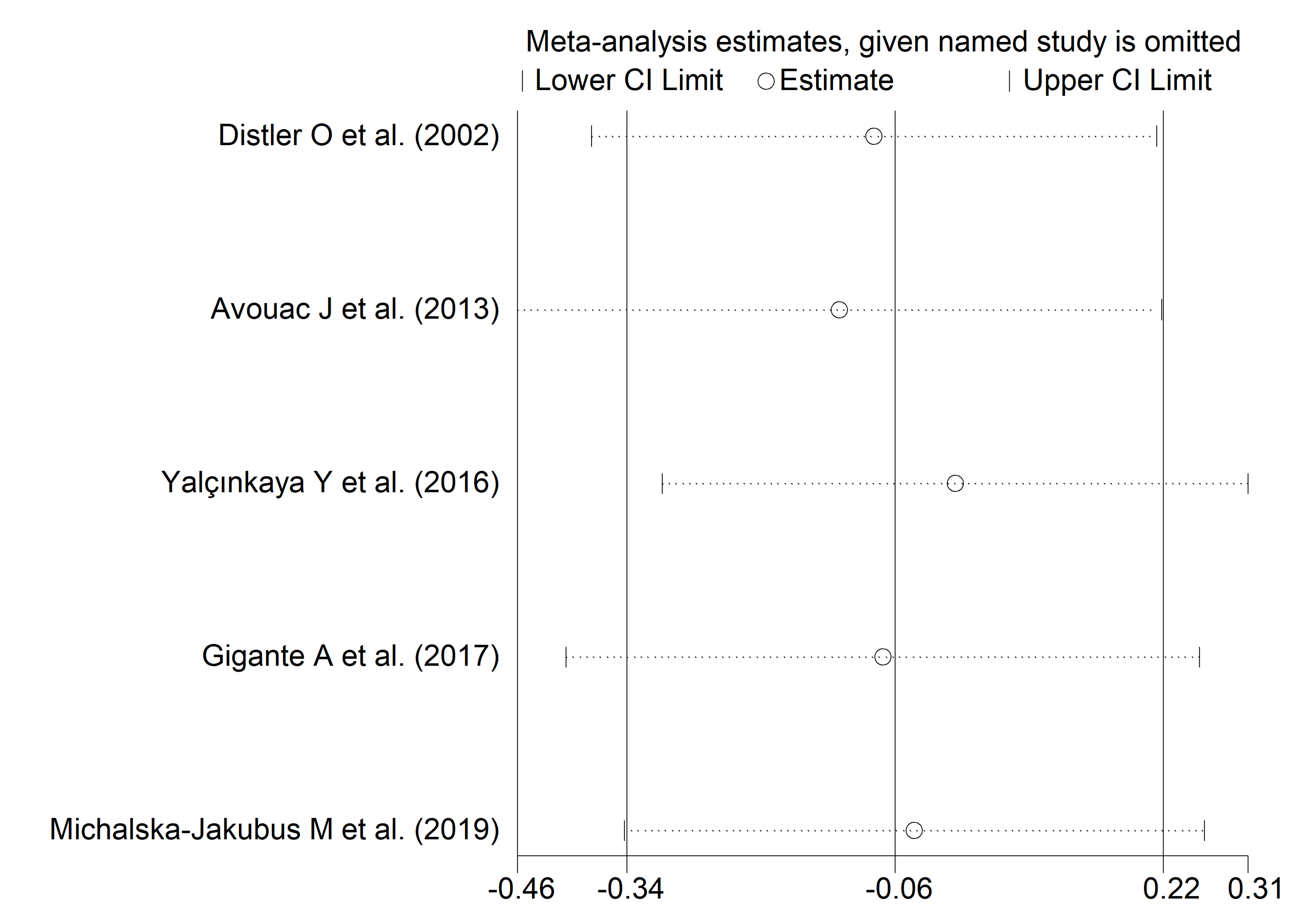

Supplement: Supplementary file 6 [file Image6.tif]

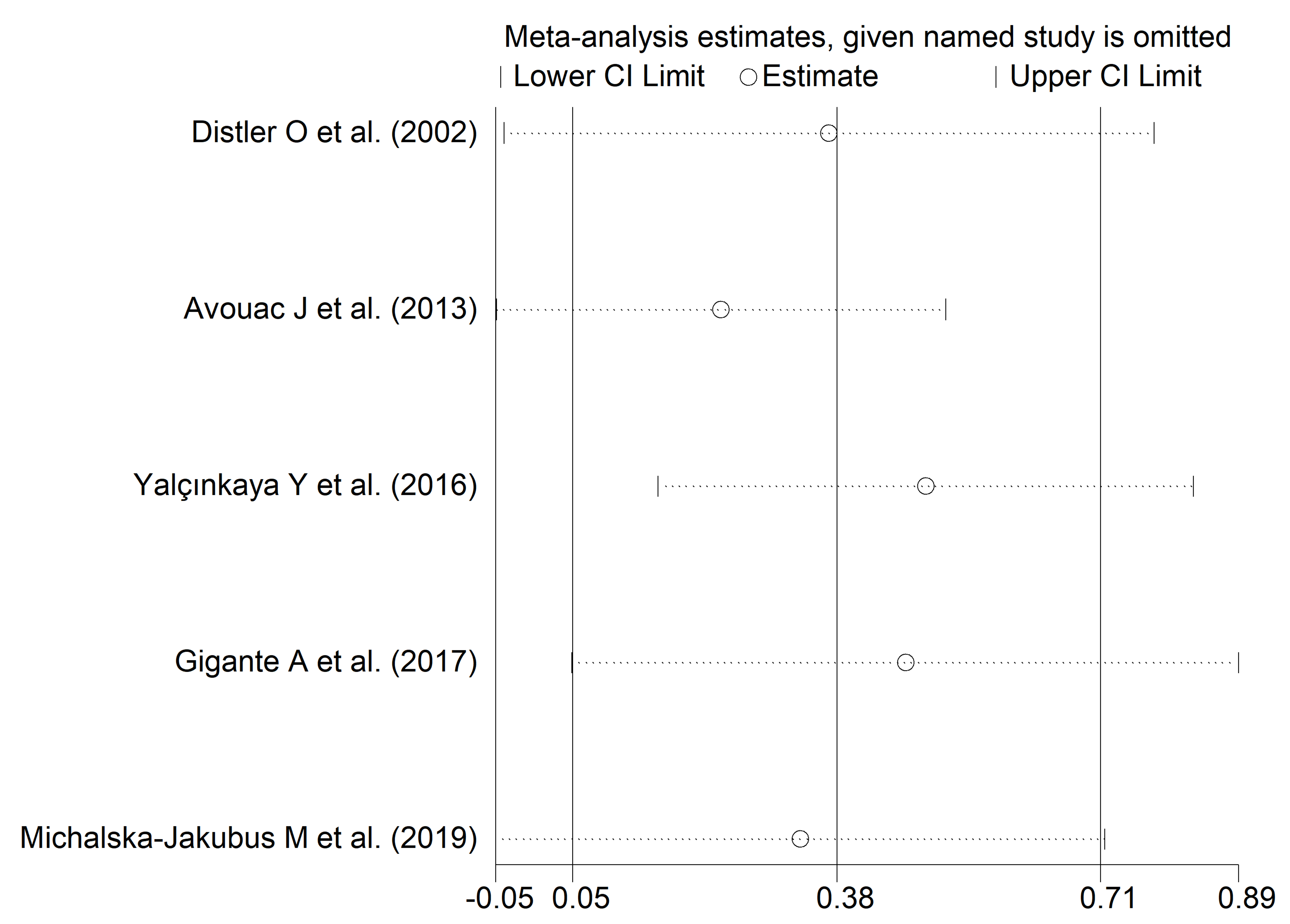

Supplement: Supplementary file 7 [file Image7.tif]

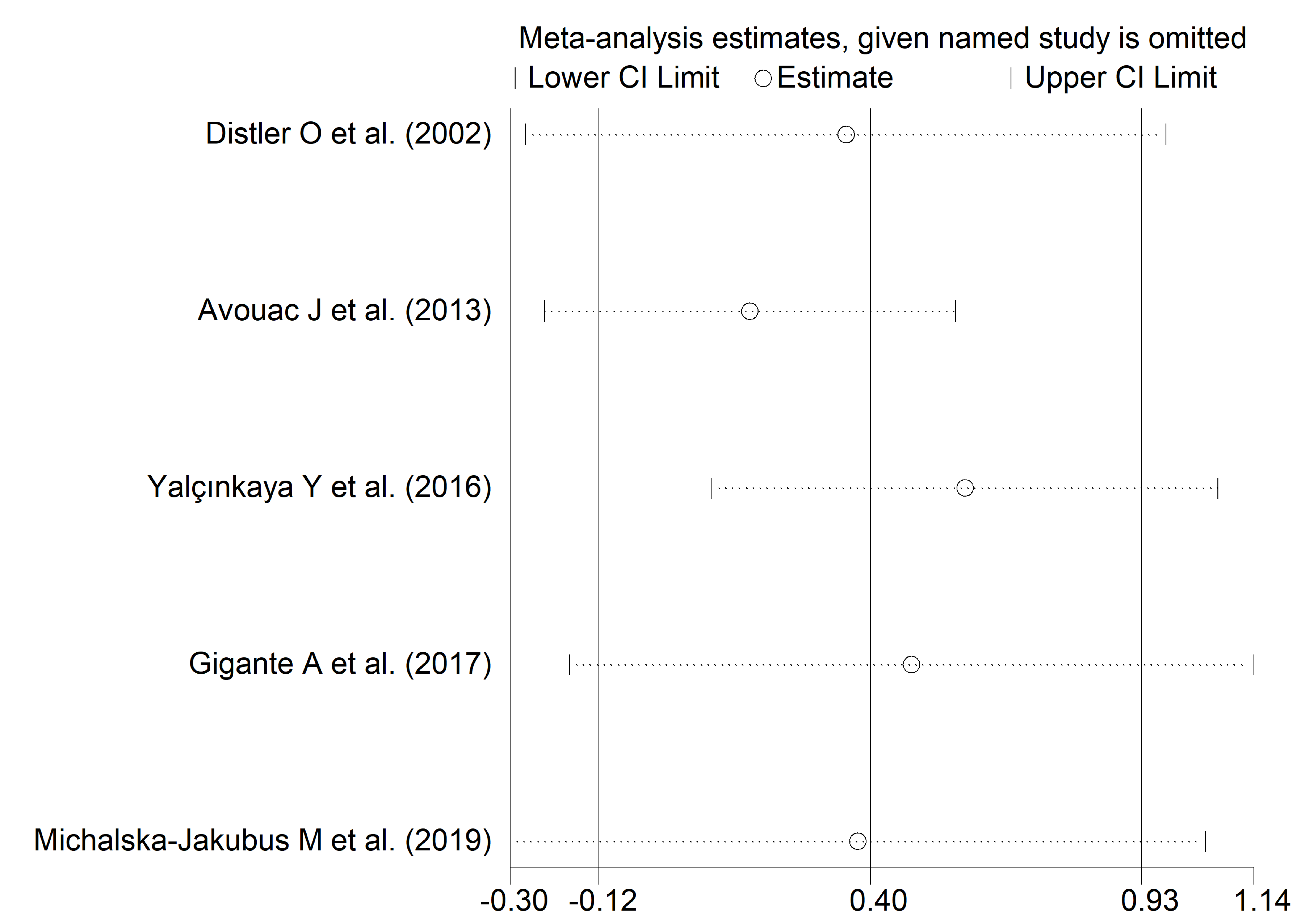

Supplement: Supplementary file 8 [file Image8.tif]

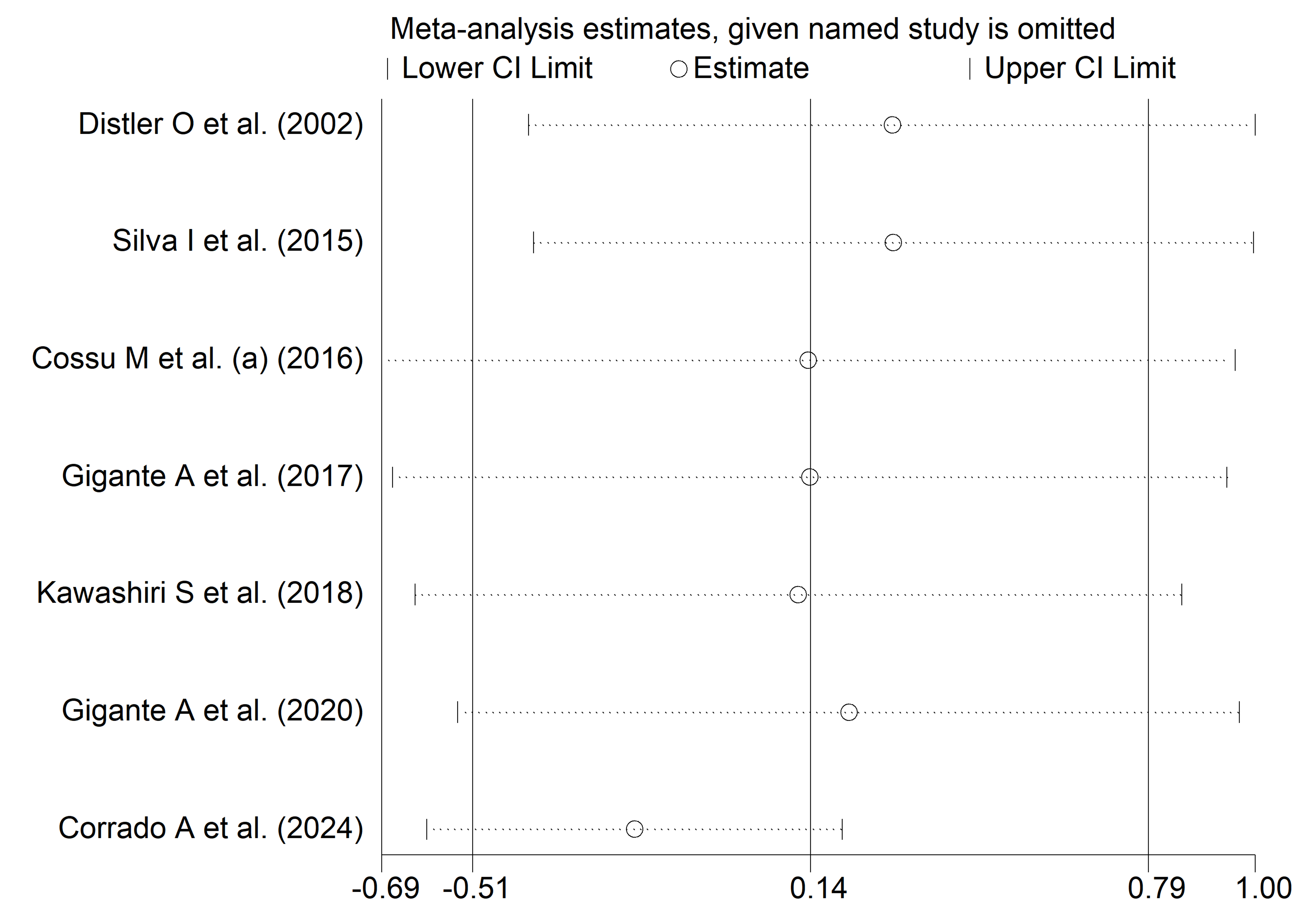

Supplement: Supplementary file 9 [file Image9.tif]

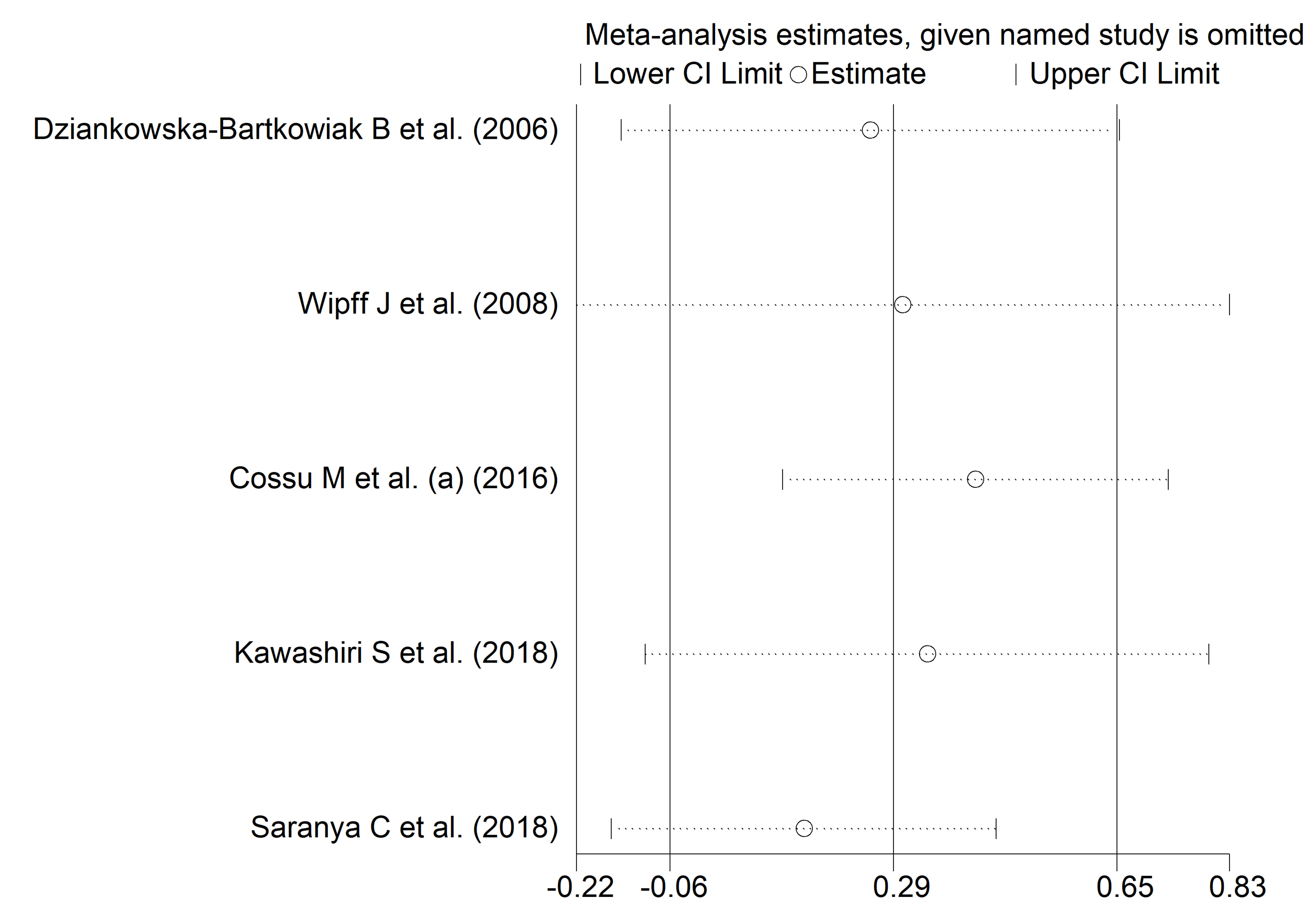

Supplement: Supplementary file 10 [file Image10.tif]
