# Supplementary material for: Vascular endothelial growth factor as a potential biomarker in systemic sclerosis: a systematic review and meta-analysis
Source: Front Immunol. 2024 Nov 28;15:1442913. doi: 10.3389/fimmu.2024.1442913 (PMC11634811; doi:10.3389/fimmu.2024.1442913)
Supplement: Supplementary file 12 [file Table2.docx]

**Supplementary Table 2.** Assessment of the risk of bias.

| **Study** | **Were the inclusion criteria clearly defined?** | **Were the subjects and the setting described in detail?** | **Was the exposure measured in a reliable way?** | **Were standard criteria used to assess the condition?** | **Were confounding factors identified?** | **Were strategies to deal with confounding factors stated?** | **Were the outcomes measured in a reliable way?** | **Was appropriate statistical analysis used?** | **Risk of bias** |
| --- | --- | --- | --- | --- | --- | --- | --- | --- | --- |
| Harada M et al. (1) | No | No | Yes | Yes | No | No | Yes | Yes | Moderate |
| Kikuchi K et al. (2) | No | No | Yes | Yes | No | No | Yes | Yes | Moderate |
| Sato S et al. (3) | No | No | Yes | Yes | Yes | Yes | Yes | Yes | Low |
| Distler O et al. (4) | Yes | Yes | Yes | Yes | No | No | Yes | Yes | Low |
| Choi JJ et al. (5) | No | Yes | Yes | Yes | No | No | Yes | Yes | Moderate |
| Hashimoto N et al. (6) | No | No | Yes | Yes | No | No | Yes | Yes | Moderate |
| Allanore Y et al. (7) | Yes | Yes | Yes | Yes | No | No | Yes | Yes | Low |
| Kuwana M et al. (8) | Yes | Yes | Yes | Yes | No | No | Yes | Yes | Low |
| Kuryliszyn-Moskal A et al. (9) | No | Yes | Yes | Yes | No | No | Yes | Yes | Moderate |
| Dziankowska-Bartkowiak B et al. (10) | Yes | Yes | Yes | Yes | No | No | Yes | Yes | Low |
| Wipff J et al. (11) | No | Yes | Yes | Yes | No | No | Yes | Yes | Moderate |
| Hummers LK et al. (12) | No | Yes | Yes | Yes | Yes | Yes | Yes | Yes | Low |
| Papaioannou AI et al. (13) | Yes | Yes | Yes | Yes | Yes | Yes | Yes | Yes | Low |
| Solanilla A et al. (14) | No | Yes | Yes | Yes | No | No | Yes | Yes | Moderate |
| Distler JHW et al. (15) | No | Yes | Yes | Yes | Yes | Yes | Yes | Yes | Low |
| Riccieri V et al. (16) | No | Yes | Yes | Yes | Yes | Yes | Yes | Yes | Low |
| Avouac J et al. (17) | No | Yes | Yes | Yes | Yes | Yes | Yes | Yes | Low |
| Aydoğdu E et al. (18) | Yes | Yes | Yes | Yes | No | No | Yes | Yes | Low |
| Farouk HM et al. (19) | No | Yes | Yes | Yes | No | No | Yes | Yes | Moderate |
| Koca SS et al. (20) | No | No | Yes | Yes | No | No | Yes | Yes | Moderate |
| Reiseter S et al. (21) | No | Yes | Yes | Yes | Yes | Yes | Yes | Yes | Low |
| Silva I et al. (22) | Yes | Yes | Yes | Yes | Yes | Yes | Yes | Yes | Low |
| Cossu M et al. (23) | No | Yes | Yes | Yes | No | No | Yes | Yes | Moderate |
| Park JK et al. (24) | No | Yes | Yes | Yes | No | No | Yes | Yes | Moderate |
| Yalçınkaya Y et al. (25) | No | Yes | Yes | Yes | No | No | Yes | Yes | Moderate |
| Benyamine A et al. (26) | No | Yes | Yes | Yes | Yes | Yes | Yes | Yes | Low |
| Shenavandeh S et al. (27) | Yes | Yes | Yes | Yes | No | No | Yes | Yes | Low |
| Ibrahim SE et al. (28) | Yes | Yes | Yes | Yes | Yes | Yes | Yes | Yes | Low |
| Saranya C et al. (29) | Yes | Yes | Yes | Yes | No | No | Yes | Yes | Low |
| Michalska-Jakubus M et al. (30) | Yes | Yes | Yes | Yes | No | No | Yes | Yes | Low |
| Gigante A et al. (31) | Yes | Yes | Yes | Yes | No | No | Yes | Yes | Low |
| LV T et al. (32) | Yes | Yes | Yes | Yes | No | No | Yes | Yes | Low |
| Waszczykowska A et al. (33) | Yes | Yes | Yes | Yes | No | No | Yes | Yes | Low |
| El Gharbawy NH et al. (34) | Yes | Yes | Yes | Yes | No | No | Yes | Yes | Low |
| Stern EP et al. (35) | No | Yes | Yes | Yes | No | No | Yes | Yes | Moderate |
| Bhattacharjee D et al. (36) | Yes | Yes | Yes | Yes | No | No | Yes | Yes | Low |
| Kosałka-Wegiel J et al. (37) | Yes | Yes | Yes | Yes | Yes | Yes | Yes | Yes | Low |
| Corrado A et al. (38) | Yes | No | Yes | Yes | Yes | Yes | Yes | Yes | Low |
| Kawashiri S et al. (39) | No | Yes | No | Yes | Yes | Yes | Yes | Yes | Low |
| Gigante A et al. (40) | Yes | Yes | Yes | Yes | No | No | Yes | Yes | Low |
| Jouvray M et al. (41) | No | Yes | Yes | Yes | Yes | Yes | Yes | Yes | Low |
| De Santis M et al. (42) | No | Yes | Yes | Yes | Yes | Yes | Yes | Yes | Low |

**References**

1. Harada M, Mitsuyama K, Yoshida H, Sakisaka S, Taniguchi E, Kawaguchi T, et al. Vascular endothelial growth factor in patients with rheumatoid arthritis. Scand J Rheumatol. 1998;27(5):377-80. doi: 10.1080/03009749850154429

2. Kikuchi K, Kubo M, Kadono T, Yazawa N, Ihn H, Tamaki K. Serum concentrations of vascular endothelial growth factor in collagen diseases. Br J Dermatol. 1998;139(6):1049-51. doi: 10.1046/j.1365-2133.1998.02563.x

3. Sato S, Hasegawa M, Takehara K. Serum levels of interleukin-6 and interleukin-10 correlate with total skin thickness score in patients with systemic sclerosis. J Dermatol Sci. 2001;27(2):140-6. doi: 10.1016/s0923-1811(01)00128-1

4. Distler O, del Rosso A, Giacomelli R, Cipriani P, Conforti ML, Guiducci S, et al. Angiogenic and angiostatic factors in systemic sclerosis: increased levels of vascular endothelial growth factor are a feature of the earliest disease stages and are associated with the absence of fingertip ulcers. Arthritis Research & Therapy. 2002;4(6). doi: 10.1186/ar596

5. Choi JJ, Min DJ, Cho ML, Min SY, Kim SJ, Lee SS, et al. Elevated vascular endothelial growth factor in systemic sclerosis. J Rheumatol. 2003;30(7):1529-33. doi:

6. Hashimoto N, Iwasaki T, Kitano M, Ogata A, Hamano T. Levels of vascular endothelial growth factor and hepatocyte growth factor in sera of patients with rheumatic diseases. Modern Rheumatology. 2003;13(2):129-34. doi: 10.3109/s10165-002-0211-8

7. Allanore Y, Borderie D, Lemarechal H, Ekindjian OG, Kahan A. Nifedipine decreases sVCAM-1 concentrations and oxidative stress in systemic sclerosis but does not affect the concentrations of vascular endothelial growth factor or its soluble receptor 1. Arthritis Res Ther. 2004;6(4):R309-14. doi: 10.1186/ar1183

8. Kuwana M, Okazaki Y, Yasuoka H, Kawakami Y, Ikeda Y. Defective vasculogenesis in systemic sclerosis. The Lancet. 2004;364(9434):603-10. doi: 10.1016/s0140-6736(04)16853-0

9. Kuryliszyn-Moskal A, Klimiuk PA, Sierakowski S. Soluble adhesion molecules (sVCAM-1, sE-selectin), vascular endothelial growth factor (VEGF) and endothelin-1 in patients with systemic sclerosis: relationship to organ systemic involvement. Clinical Rheumatology. 2005;24(2):111-6. doi: 10.1007/s10067-004-0987-3

10. Dziankowska-Bartkowiak B, Waszczykowska E, Dziankowska-Zaboroszczyk E, de Graft-Johnson JE, Zalewska A, Luczynska M, et al. Decreased ratio of circulatory vascular endothelial growth factor to endostatin in patients with systemic sclerosis--association with pulmonary involvement. Clin Exp Rheumatol. 2006;24(5):508-13. doi:

11. Wipff J, Avouac J, Borderie D, Zerkak D, Lemarechal H, Kahan A, et al. Disturbed angiogenesis in systemic sclerosis: high levels of soluble endoglin. Rheumatology. 2008;47(7):972-5. doi: 10.1093/rheumatology/ken100

12. Hummers LK, Hall AMY, Wigley FM, Simons M. Abnormalities in the Regulators of Angiogenesis in Patients with Scleroderma. The Journal of Rheumatology. 2009;36(3):576-82. doi: 10.3899/jrheum.080516

13. Papaioannou AI, Zakynthinos E, Kostikas K, Kiropoulos T, Koutsokera A, Ziogas A, et al. Serum VEGF levels are related to the presence of pulmonary arterial hypertension in systemic sclerosis. BMC Pulmonary Medicine. 2009;9(1). doi: 10.1186/1471-2466-9-18

14. Solanilla A, Villeneuve J, Auguste P, Hugues M, Alioum A, Lepreux S, et al. The transport of high amounts of vascular endothelial growth factor by blood platelets underlines their potential contribution in systemic sclerosis angiogenesis. Rheumatology. 2009;48(9):1036-44. doi: 10.1093/rheumatology/kep154

15. Distler JHW, Strapatsas T, Huscher D, Dees C, Akhmetshina A, Kiener HP, et al. Dysbalance of angiogenic and angiostatic mediators in patients with mixed connective tissue disease. Annals of the Rheumatic Diseases. 2011;70(7):1197-202. doi: 10.1136/ard.2010.140657

16. Riccieri V, Stefanantoni K, Vasile M, Macri V, Sciarra I, Iannace N, et al. Abnormal plasma levels of different angiogenic molecules are associated with different clinical manifestations in patients with systemic sclerosis. Clin Exp Rheumatol. 2011;29(2 Suppl 65):S46-52. doi:

17. Avouac J, Vallucci M, Smith V, Senet P, Ruiz B, Sulli A, et al. Correlations between angiogenic factors and capillaroscopic patterns in systemic sclerosis. Arthritis Research & Therapy. 2013;15(2). doi: 10.1186/ar4217

18. Aydoğdu E, Pamuk ÖN, Dönmez S, Pamuk GE. Decreased interleukin-20 level in patients with systemic sclerosis: are they related with angiogenesis? Clinical Rheumatology. 2013;32(11):1599-603. doi: 10.1007/s10067-013-2317-0

19. Farouk HM, Hamza SH, El Bakry SA, Youssef SS, Aly IM, Moustafa AA, et al. Dysregulation of angiogenic homeostasis in systemic sclerosis. Int J Rheum Dis. 2013;16(4):448-54. doi: 10.1111/1756-185X.12130

20. Koca SS, Akbas F, Ozgen M, Yolbas S, Ilhan N, Gundogdu B, et al. Serum galectin-3 level in systemic sclerosis. Clinical Rheumatology. 2013;33(2):215-20. doi: 10.1007/s10067-013-2346-8

21. Reiseter S, Molberg Ø, Gunnarsson R, Lund MB, Aalokken TM, Aukrust P, et al. Associations between circulating endostatin levels and vascular organ damage in systemic sclerosis and mixed connective tissue disease: an observational study. Arthritis Research & Therapy. 2015;17(1). doi: 10.1186/s13075-015-0756-5

22. Silva I, Teixeira A, Oliveira J, Almeida I, Almeida R, Vasconcelos C. Predictive value of vascular disease biomarkers for digital ulcers in systemic sclerosis patients. Clin Exp Rheumatol. 2015;33(4 Suppl 91):S127-30. doi:

23. Cossu M, Andracco R, Santaniello A, Marchini M, Severino A, Caronni M, et al. Serum levels of vascular dysfunction markers reflect disease severity and stage in systemic sclerosis patients. Rheumatology. 2016;55(6):1112-6. doi: 10.1093/rheumatology/kew017

24. Park JK, Fava A, Carrino J, Del Grande F, Rosen A, Boin F. Association of Acroosteolysis With Enhanced Osteoclastogenesis and Higher Blood Levels of Vascular Endothelial Growth Factor in Systemic Sclerosis. Arthritis & Rheumatology. 2016;68(1):201-9. doi: 10.1002/art.39424

25. Yalçınkaya Y, Adın- Çınar S, Artim-Esen B, Kamalı S, Pehlivan Ö, Öcal L, et al. Capillaroscopic findings and vascular biomarkers in systemic sclerosis: Association of low CD40L levels with late scleroderma pattern. Microvascular Research. 2016;108:17-21. doi: 10.1016/j.mvr.2016.07.002

26. Benyamine A, Magalon J, Cointe S, Lacroix R, Arnaud L, Bardin N, et al. Increased serum levels of fractalkine and mobilisation of CD34+CD45− endothelial progenitor cells in systemic sclerosis. Arthritis Research & Therapy. 2017;19(1). doi: 10.1186/s13075-017-1271-7

27. Shenavandeh S, Tarakemeh T, Sarvestani EK, Nazarinia MA. Serum vascular endothelial growth factor (VEGF), soluble VEGF receptor-1 (sVEGFR-1) and sVEGFR-2 in systemic sclerosis patients: Relation to clinical manifestations and capillaroscopy findings. The Egyptian Rheumatologist. 2017;39(1):19-24. doi: 10.1016/j.ejr.2016.03.004

28. Ibrahim SE, Morad CS, Farouk N, Louis A. Platelet indices as markers of inflammation in systemic sclerosis patients: Relation to vascular endothelial growth factor and flow mediated dilatation. The Egyptian Rheumatologist. 2018;40(4):239-42. doi: 10.1016/j.ejr.2017.12.001

29. Saranya C, Ramesh R, Bhuvanesh M, Balaji C, Balameena S, Rajeswari S. Serum vascular endothelial growth factor levels as a marker of skin thickening, digital ischemia, and interstitial lung disease in systemic sclerosis. Indian Journal of Rheumatology. 2018;13(3). doi: 10.4103/injr.injr_132_17

30. Michalska-Jakubus M, Cutolo M, Smith V, Krasowska D. Imbalanced serum levels of Ang1, Ang2 and VEGF in systemic sclerosis: Integrated effects on microvascular reactivity. Microvascular Research. 2019;125. doi: 10.1016/j.mvr.2019.103881

31. Gigante A, Gasperini ML, Rosato E, Navarini L, Margiotta D, Afeltra A, et al. Phase angle could be a marker of microvascular damage in systemic sclerosis. Nutrition. 2020;73. doi: 10.1016/j.nut.2020.110730

32. Lv T, Yang F, Zhang K, Lv M, Zhang Y, Zhu P. The risk of circulating angiogenic T cells and subsets in patients with systemic sclerosis. International Immunopharmacology. 2020;81. doi: 10.1016/j.intimp.2020.106282

33. Waszczykowska A, Goś R, Waszczykowska E, Dziankowska-Bartkowiak B, Podgórski M, Jurowski P. The Role of Angiogenesis Factors in the Formation of Vascular Changes in Scleroderma by Assessment of the Concentrations of VEGF and sVEGFR2 in Blood Serum and Tear Fluid. Mediators of Inflammation. 2020;2020:1-8. doi: 10.1155/2020/7649480

34. El Gharbawy NH, Sheha DS, Bawady SA, El Leithy SA. Vascular endothelial growth factor profile and Vitamin D level in Systemic Sclerosis Egyptian patients. Egypt J Immunol. 2021;28(3):168-75. doi:

35. Stern EP, Unwin R, Burns A, Ong VH, Denton CP. Exploring molecular pathology of chronic kidney disease in systemic sclerosis by analysis of urinary and serum proteins. Rheumatology Advances in Practice. 2021;5(1). doi: 10.1093/rap/rkaa083

36. Bhattacharjee D, Mondal S, Saha A, Misra S, Chatterjee S, Rao A, et al. Effect of vasodilator and immunosuppressive therapy on the endothelial dysfunction in patients with systemic sclerosis. Clinical and Experimental Medicine. 2022;23(3):905-15. doi: 10.1007/s10238-022-00845-w

37. Kosałka-Węgiel J, Lichołai S, Dziedzina S, Milewski M, Kuszmiersz P, Korona A, et al. Association between clinical features and course of systemic sclerosis and serum interleukin-8, vascular endothelial growth factor, basic fibroblast growth factor, and interferon alpha. Advances in Clinical and Experimental Medicine. 2023;33(4):369-77. doi: 10.17219/acem/168724

38. Corrado A, Mansueto N, Correale M, Rella V, Tricarico L, Altomare A, et al. Flow Mediated Dilation in Systemic Sclerosis: Association with clinical findings, capillaroscopic patterns and endothelial circulating markers. Vascular Pharmacology. 2024;154. doi: 10.1016/j.vph.2023.107252

39. Kawashiri SY, Nishino A, Igawa T, Takatani A, Shimizu T, Umeda M, et al. Prediction of organ involvement in systemic sclerosis by serum biomarkers and peripheral endothelial function. Clin Exp Rheumatol. 2018;36 Suppl 113(4):102-8. doi:

40. Gigante A, Navarini L, Margiotta D, Amoroso A, Barbano B, Cianci R, et al. Angiogenic and angiostatic factors in renal scleroderma-associated vasculopathy. Microvascular Research. 2017;114:41-5. doi: 10.1016/j.mvr.2017.06.003

41. Jouvray M, Launay D, Dubucquoi S, Sobanski V, Podevin C, Lambert M, et al. Whole-Body Distribution and Clinical Association of Telangiectases in Systemic Sclerosis. JAMA Dermatology. 2018;154(7). doi: 10.1001/jamadermatol.2018.0916

42. De Santis M, Bosello SL, Capoluongo E, Inzitari R, Peluso G, Lulli P, et al. A vascular endothelial growth factor deficiency characterises scleroderma lung disease. Annals of the Rheumatic Diseases. 2012;71(9):1461-5. doi: 10.1136/annrheumdis-2011-200657
