# Supplementary material for: Vascular endothelial growth factor as a potential biomarker in systemic sclerosis: a systematic review and meta-analysis
Source: Front Immunol. 2024 Nov 28;15:1442913. doi: 10.3389/fimmu.2024.1442913 (PMC11634811; doi:10.3389/fimmu.2024.1442913)
Supplement: Supplementary file 13 [file Table3.docx]

**Supplementary figure legends**

**Supplementary Figure 1.** Sensitivity analysis of the association between VEGF concentrations and SSc.

**Supplementary Figure 2.** Funnel plot of studies investigating the association between VEGF concentrations and SSc after “trimming-and-filling”. Dummy studies and genuine studies are represented by enclosed circles and free circles, respectively.

**Supplementary Figure 3.** Sensitivity analysis of the association between VEGF concentrations and disease form.

**Supplementary Figure 4.** Funnel plot of studies investigating the association between VEGF concentrations and disease form after “trimming-and-filling”. Dummy studies and genuine studies are represented by enclosed circles and free circles, respectively.

**Supplementary Figure 5.** Bubble plot reporting univariate meta-regression analysis between effect size and publication year (A) and cumulative meta-analysis of VEGF concentrations based on publication year (B).

**Supplementary Figure 6.** Sensitivity analysis of the association between VEGF concentrations and capillaroscopy pattern (early vs. active).

**Supplementary Figure 7.** Sensitivity analysis of the association between VEGF concentrations and capillaroscopy pattern (active vs. late).

**Supplementary Figure 8.** Sensitivity analysis of the association between VEGF concentrations and capillaroscopy pattern (early vs. late).

**Supplementary Figure 9.** Sensitivity analysis of the association between VEGF concentrations and digital ulcers.

**Supplementary Figure 10.** Sensitivity analysis of the association between VEGF concentrations and interstitial lung disease.
